# SOX17 enhancer variants disrupt transcription factor binding and enhancer inactivity drives pulmonary hypertension

**DOI:** 10.1161/CIRCULATIONAHA.122.061940

**Published:** 2023-04-17

**Authors:** Rachel Walters, Eleni Vasilaki, Jurjan Aman, Chien-Nien Chen, Yukyee Wu, Olin D Liang, Ali Ashek, Olivier Dubois, Lin Zhao, Farah Sabrin, Inês Cebola, Jorge Ferrer, Nicholas W Morrell, James R Klinger, Martin R Wilkins, Lan Zhao, Christopher J Rhodes

**Affiliations:** 1 National Heart and Lung Institute, Hammersmith Hospital, Imperial College, London, UK; 2 Dept. of Pulmonary Medicine, Amsterdam University Medical Center, Amsterdam, The Netherlands; 3 Division of Hematology/Oncology, Department of Medicine, Rhode Island Hospital and Warren Alpert Medical School of Brown University, Providence, RI, USA; 4 Section of Genetics & Genomics, Department of Metabolism, Digestion & Reproduction, Hammersmith Hospital, Imperial College, London, UK; 5 Computational Biology and Health Genomics Programme, Centre for Genomic Regulation (CRG), The Barcelona Institute of Science and Technology (BIST), Barcelona, Spain; 6 Centro de Investigación Biomédica en Red de Diabetes y Enfermedades Metabólicas Asociadas (CIBERDEM), Barcelona, Spain; 7 Department of Medicine, University of Cambridge, Cambridge, UK; 8 NIHR BioResource for Translational Research, University of Cambridge, Cambridge, UK; 9 On Behalf of the British Heart Foundation/Medical Research Council UK PAH Cohort Consortium; 10 Division of Pulmonary, Sleep and Critical Care Medicine, Department of Medicine, Rhode Island Hospital, Warren Alpert Medical School of Brown University, Providence, RI, 02806, USA

**Keywords:** Pulmonary Hypertension, PH, SOX17

## Abstract

**Background:**

Pulmonary arterial hypertension (PAH) is a rare disease characterised by remodelling of the pulmonary arteries, increased vascular resistance and right heart failure. Genome-wide association studies (GWAS) of idiopathic/heritable PAH established novel genetic risk variants including conserved enhancers upstream of transcription factor (TF) *SOX17* containing two independent signals. SOX17 is an important transcription factor in embryonic development and in the homeostasis of pulmonary artery endothelial cells (hPAEC) in the adult. Rare pathogenic mutations in *SOX17* cause heritable PAH. We hypothesised that PAH risk alleles in an enhancer region impair TF-binding upstream of *SOX17*, which in turn reduces *SOX17* expression and contributes to disturbed endothelial cell function and PAH development.

**Methods:**

CRISPR manipulation and small interfering RNA were used to modulate *SOX17* expression. Electromobility shift assays (EMSA) were used to confirm *in-silico*-predicted TF differential binding to the *SOX17* variants. Functional assays in hPAEC were used to establish the biological consequences of *SOX17* loss. *In-silico* analysis using the connectivity map (CMap) were used to predict compounds that rescue disturbed *SOX17* signalling. Mice with deletion of the *SOX17* signal 1 enhancer region (*SOX17*-4593/enhKO) were phenotyped in response to chronic hypoxia and SU5416/hypoxia.

**Results:**

CRISPR-Inhibition of *SOX17*-signal 2 and deletion of *SOX17*-signal 1 specifically decreased *SOX17* expression. EMSA demonstrated differential binding of hPAEC nuclear proteins to the risk and non-risk alleles from both *SOX17* signals. Candidate TFs HOXA5 and ROR-α were identified through in silico analysis and antibody EMSA. Analysis of the hPAEC transcriptomes revealed alteration of PAH-relevant pathways upon *SOX17* silencing, including extracellular matrix regulation. *SOX17* silencing in hPAEC resulted in increased apoptosis, proliferation, and disturbance of barrier function. Using CMap, compounds were identified that reversed the SOX17-dysfunction transcriptomic signatures in hPAECs. SOX17 enhancer knockout in mice reduced lung SOX17 expression, resulting in more severe pulmonary vascular leak and hypoxia or SU5416/hypoxia-induced pulmonary hypertension.

**Conclusions:**

Common PAH risk variants upstream of the *SOX17* promoter reduce endothelial *SOX17* expression, at least in part, through differential binding of HOXA5 and ROR-α. Reduced SOX17 expression results in disturbed hPAEC function and PAH. Existing drug compounds can reverse the disturbed SOX17 pulmonary endothelial transcriptomic signature.

## Introduction

Pulmonary arterial hypertension (PAH) is a rare but lethal disease. With no intervention, the mean survival is 2.8 years (1) and with modern therapeutic intervention, the rate of mortality in the first year is around 15% (2). Increased pulmonary vascular resistance in PAH is driven by vasoconstriction, inflammation, and proliferative remodelling of the intima and media of precapillary arteries (3,4). The endothelium of healthy pulmonary arteries forms a semi-permeable barrier, which dynamically adapts to external stimuli such as shear stress or hypoxia. Injury or dysfunction of the endothelium is thought to be an early, yet poorly understood, trigger in PAH development. While genetic factors enhance susceptibility (e.g. bone morphogenetic protein receptor 2, *BMPR2* variants), environmental factors like hypoxia, change in shear stress, inflammation, drugs or toxins can directly injure the endothelial barrier, leading to apoptosis, loss of barrier integrity and vascular remodelling of the pulmonary artery wall (5).

Rare pathogenic variants in several genes, most commonly *BMPR2,* are associated with PAH (6), but ˜75% of idiopathic cases cannot be explained by these variants. A recent large genome-wide association study (GWAS), using data from 11,744 European individuals (2,085 patients) identified two independent PAH risk variant-containing signals (*SOX17*-signal 1 and *SOX17*-signal 2) in a region located 106-200kb upstream of the *SOX17* gene promoter. The risk alleles are common in the populations tested and enriched in PAH, with 59% of patients homozygous for the risk allele of both signals compared to 46% of controls (7). In addition, whole genome sequencing studies identified rare deleterious variants in the *SOX17* gene associated with the development of severe PAH. Therefore, *SOX17* has the potential to provide a powerful insight into PAH risk via rare and common variants.

The *SOX17* gene encodes the transcription factor, SOX17, which is a member of the SoxF protein subfamily. SoxF proteins are important regulators of cell fate and differentiation (8) and have key roles in cardiovascular development (9). SOX17 is essential for developmental angiogenesis and arterial differentiation in the embryo. In the adult, SOX17 plays a role in maintaining arterial identity and tumor angiogenesis (10,11). EC deletion of SOX17 in mouse models leads to embryonic lethality due to underdeveloped arteries and a complete lack of large arteries (10). Conditional deletion of *SOX17* in splanchnic mesenchyme-derivatives leads to severe vascular abnormalities, including reduced branching of pulmonary arties and dilated cardiomyopathy (12). Thus far, the role of SOX17 in the human pulmonary arterial endothelium remains unclear. In addition, it remains unclear how upstream common variants increase the risk of PAH. Variation in *SOX17*-signal 1 has been shown to affect *SOX17* expression, but the cellular and in vivo function of this element is still poorly understood (7); clear function has not been defined for *SOX17*-signal 2 region to date. We hypothesised the PAH variants upstream of *SOX17* drive allele-specific transcription factor (TF) binding at the two signals which affects *SOX17* expression, hPAEC function and PAH development.

## Methods

For full methods and materials please see the supplemental section. We have deposited the RNAseq data to GEO with accession number GSE214742. All other data are available upon reasonable request.

### Patient endothelial cells

Individuals with a diagnosis of idiopathic PAH (n=11) diagnosed according to international guidelines (13) and healthy controls who did not self-report cardiovascular or respiratory conditions (n=5) were recruited between 23/Aug/2017 and 18/Sep/2019 from the National Pulmonary Hypertension service and staff at Hammersmith Hospital for derivation of endothelial colony forming cells (ECFC) from blood. Samples were obtained with written, informed consent and local research ethics committee approval. ECFC were isolated and cultured as previously described: (14) and extracted for RNA following growth in 2% FBS-supplemented EGM-2 media plus indicated treatments or vehicle for 24 hours.

### CRISPR-Manipulation

To inhibit *SOX17*-signal 2 and -signal 1, CRISPR−Inhibition (CRISPR-I) was used as previously described (7). To delete *SOX17*-signal 1, CRISPR-Deletion (CRISPR-D) was used. Two guides (Table S1) against *SOX17*-signal 1 were cloned into a pSpCas9(BB)-T2A-HygR vector (#118153) using a one-step dual CRISPR/Cas9 guide RNA cloning protocol as previously described (15).

### EMSA & Supershift

To investigate transcription factors (TF) whose binding may be affected due to the *SOX17* variants, using TF databases CIS−BP, PROMO and ConSite were used. To investigate the differential binding of TFs to the risk and non-risk allele present at the *SOX17* variants, an EMSA was performed with the LightShift (chemiluminescent) EMSA kit according to manufacturer’s instructions. To investigate which transcription factor binds to the *SOX17* variants, supershift assays with anti-HOXa5/RORa antibodies were performed. Five hPAEC donors were also used for chromatin immunoprecipitation (ChIP) qPCR assays with the same antibodies.

### siRNA

To assess the role of *SOX17* in hPAECs, siRNA experiments were performed using silencer select siRNA (ThermoFisher) targeting *SOX17* (#s34626). Comparable control siRNA was also used and included two scrambled-siRNA (#ASO2FOQH, #4390847) and a control targeting GAPDH (#ASO2FLIC).

### RNAseq Analysis

To assess whole transcriptomic effects of knockdown of *SOX17* by siRNA and CRISPR-I *SOX17*-signal 2 and -signal 1, RNA sequencing (RNAseq) was carried out by the Imperial College BRC Genomics Facility and analysis of the dataset was performed in RStudio. To assess gene ontology changes resulting from either siRNA-*SOX17*, CRISPR-I *SOX17*-signal 2 or signal 1, over-representation analysis was performed using the WEB-based Gene SeTAnaLysis Toolkit (Webgestalt).

### qPCR

To investigate the change in expression of target genes, reverse transcription-PCR (RTPCR) and qPCR was performed with actin beta (ACTβ) used as a reference gene (2^-deltaCt^).

### Western Blotting

To assess the levels of SOX17 protein following siRNA transduction, total protein was extracted from cells using RIPA buffer (10X, Sigma) supplemented with protease and phosphatase inhibitor cocktail (ThermoFisher) and immunoblotted with anti-SOX17 (ab224637, Abcam, 1:500).

### Proteomic analysis

SomaLogic SomaScan measurements were available from a recent proteomics study by Rhodes et al (16) Patient characteristics are shown in Table S2. Peripheral venous blood was collected during patients’ routine clinical appointments.

The SNP genotypes for *SOX17* signal 1 (rs13266183) were obtained from a whole genome sequencing study from the UK National Institute for Health Research BioResource (7). Linear regression models were conducted with *SOX17* signal 1 genotypes being the independent variables, protein concentrations as the dependent variables and age and sex included as covariates. The p-values from the linear regression were corrected for multiple comparisons using the false discovery rate (FDR) method. All analyses were completed in R using RStudio v1.4.1106 and the volcano plots were designed using the package “EnhancedVolcano”.

### Cellular function assays

To assess the effect of siRNA-*SOX17* on hPAEC function, assays investigating proliferation, apoptosis, cell viability, adhesion and barrier function were used and are discussed in full in the supplemental section.

### In-silico analysis using the Connectivity Map

To analyse the differential expression patterns which occur when *SOX17* expression is manipulated, RNA-sequencing of *SOX17*-signal 1 CRISPR-I hPAECs was performed as previously stated. To discover compounds that could be repurposed for the treatment of SOX17 dysfunction, the CMap was used (17). Differential gene expression lists (Table S3) were used to create queries for the three conditions, *SOX17*-promoter activation, *SOX17*-promoter repression, and *SOX17*-signal 1 repression. Candidate compounds with a tau score of over 90 or under -90 were selected. The compounds selected were Sirolimus, Aminopurvalanol-a and YK-4279. The signature of each compound was compared with the condition signature to select gene for further analysis by qPCR.

### Animal models of PH in *SOX17* enhancer knockout

*SOX17*-enhancer knockout mice generated by CRISPR-cas9 technology at the MRC Imperial College London were phenotyped blinded both in London and at Brown University, in Providence, RI, USA to either normoxia, hypoxia or combined VEGFR2 blockade SUGEN/SU5416 and hypoxia using standard measures of PH (see Supplement) and in accordance with institutional guidelines.

## Results

### PAH common variant signals rs10958403 and rs765727 identify *SOX17* enhancers

**Figure 1):**
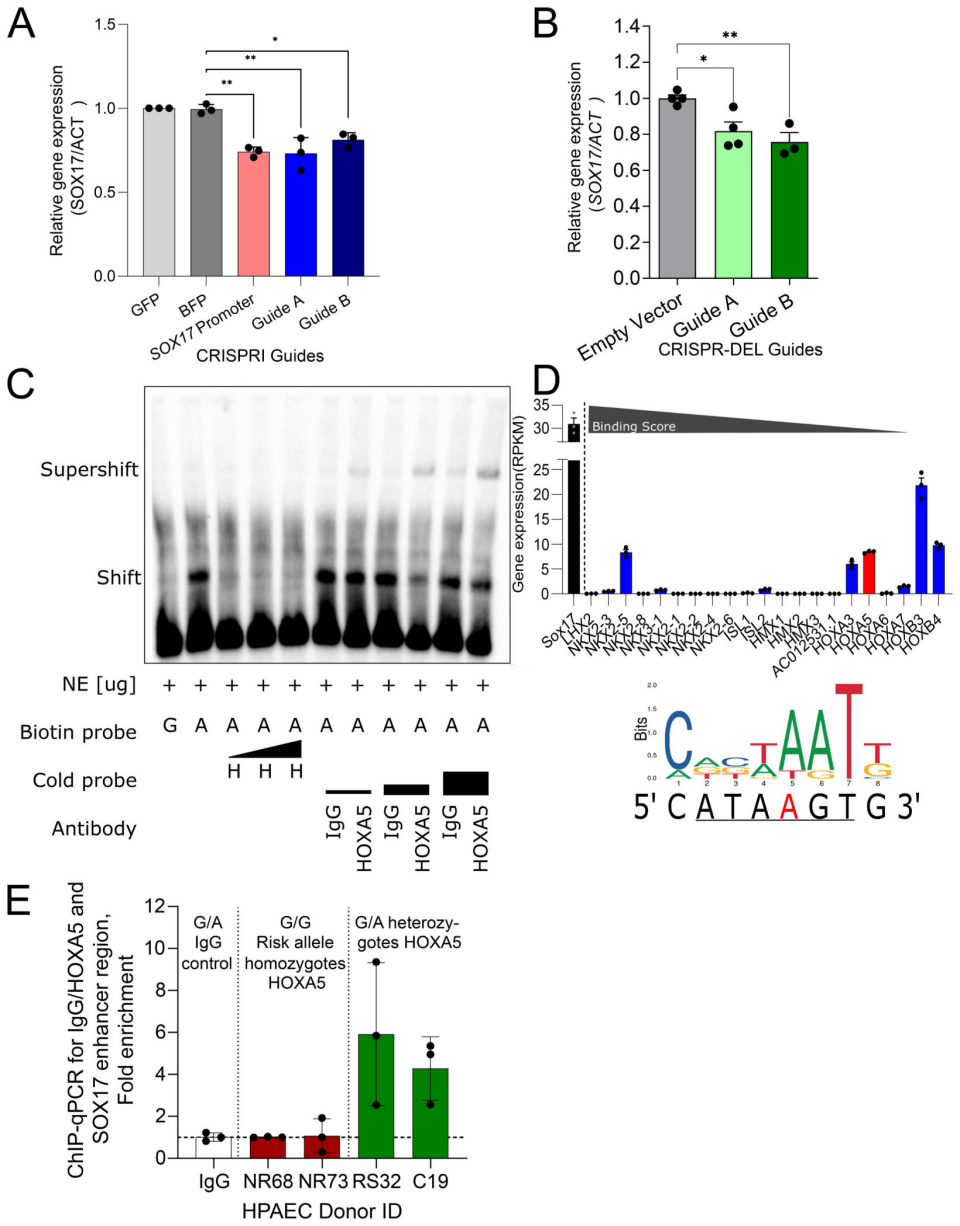
Defining Upstream Regulators of SOX17 & Effects of Common Variation at the *SOX17* Locus in PAH. **A) Knockdown of *SOX17* through CRISPR-Inhibition of *SOX17*-signal 2.** Relative gene expression of *SOX17* compared to *ACTβ* in hPAEC. Ordinary 1-way ANOVA of conditions compared to BFP condition with Dunnett’s multiple comparisons test. n=3 experiments performed in triplicate. **B) Knockdown of *SOX17* through CRISPR-deletion of *SOX17*-signal 1 region.** Relative gene expression of *SOX17* compared to *ACTβ* in hPAEC. CRISPR-deletion guides A/B target *SOX17*-signal 1. Ordinary 1-way ANOVA of conditions compared to BFP condition with Dunnett’s multiple comparisons test. n=3. **C) EMSA assay showing binding of hPAEC nuclear proteins to 21bp DNA probes containing the sequence at the rs1098403 region.** Shift and supershift are highlighted. H indicates cold probes containing a putative HOXa5 binding site. Biotin-probe, biotin-labelled probe (G/A, alleles of *SOX17* enhancer variant included in probe sequence). Cold-Probe, unlabelled probe. H, HOXa5 competitive probe. IgG, Mouse IgG. The black triangles show increasing molecular excess from left to right. **D) RNAseq expression in hPAECs (RPKM) of potential TFs of interest for rs1098403.** *HOXa5* is shown in red. *SOX17* is shown in black for reference. All other transcription factors are shown in blue and were found through CIS-BP. The binding score (taken from CIS-BP) refers to the predicted likelihood of the transcription factor binding to the given sequence and decreases from left to right. The underlined sequence refers to the potential binding location of *HOXa5* and the A in red font is the site of rs1098403. The consensus binding sequence of HOXA5 was taken from JASPAR (jaspar.genereg.net) and is shown with the sequence of the region surrounding rs1098403 and rs765727 were taken from https://genome.ucsc.edu/. *-p<0.05, **-p<0.01. n=3. **E)** **ChIP-qPCR for HOXA5 at rs10958493.** Results of a quantitative PCR (triplet measurements per donor, n=3) performed on precipitated fraction of chromatin immunoprecipitation (ChIP) with IgG or HOXa5 antibody in 4 HPAEC donors. The ChIP with IgG control was performed in HPAEC donor C19.

The function of the noncoding sequence containing rs765727 (*SOX17*-signal 2) is unknown. To test whether it is an enhancer that targets *SOX17*, we first targeted the region using CRISPR-inhibition in hPAECs. Guide RNAs targeting either rs765727 or the *SOX17* promoter led to a significant decrease in *SOX17* expression (enhancer guide A 0.73±0.055, guide B 0.81±0.025 of negative controls, both p<0.05, Figure 1A) but did not affect nearby gene *TMEM68* (Supplementary Figure 1A). One guide also decreased the expression of another nearby gene, *MRPL15* (guide B:0.83±0.013, Supplementary Figure 1A). Deletion of *SOX17*-signal 1 (rs10958403) using CRISPR-deletion guides resulted in a significant decrease in *SOX17* but had no effect on nearby genes *TMEM68* or *MRLP15* (Figure 1B, Figure S1B).

In EMSA, nuclear protein from hPAEC bound to probes representing the non-risk alleles of both *SOX17* signals, inducing a shift, but exhibited loss of binding to the risk alleles (Figure 1C, Supplementary Figure 1E). Competition EMSA of both loci showed removal of this shift with the addition of unlabelled competitive probes for the non-risk allele but not the risk allele, confirming the specificity of the protein binding to the non-risk sequences (Figure 1C, Supplementary Figure 1E).

*In silico* analyses using the TF databases CIS−BP, PROMO and ConSite, predicted multiple transcription factors more likely to bind the non-risk versus risk sequence at both *SOX17* signals (Figure 1D, Supplementary Figure 1D). These transcription factors were subsequently prioritized by the level of gene expression in hPAECs, using RNAseq, and the predicted binding score (if available). TFs with no detectable or low expression in hPAECs were not investigated further. E47 was not selected despite having the highest expression in hPAECs as a splice variant (E12) was predicted to also bind to the risk allele (C). NKX2-5 was found to be undetectable in HPAEC in alternative public databases. Of the hPAEC-expressed candidates tested (HOXa5, ROR-α, Lin54, ZFX, RAR, Figure S1) only HOXa5 and ROR-a unlabeled probes were found to compete for nuclear protein binding to the non-risk sequences. For rs10958403, a TF competition EMSA with a probe containing the HOXa5 consensus binding sequence prevented the shift seen with the non-risk allele probe. Incubation with an antibody for HOXa5 produced a supershift pattern consistent with a probe-protein-antibody complex (Figure 1C). For rs765727, a TF competition EMSA with a probe containing the ROR-α consensus sequence prevented the shift seen with the non-risk allele probe. Incubation with an antibody for ROR-α also showed removal of the shift pattern (Supplementary Figure 1E). To validate the EMSA findings a ChIP was performed using an antibody against HOXa5 in HPAEC nuclear lysates and qPCR for the area containing rs10958403. The area containing SNP rs10958403 was only amplified in donors containing a non-risk A-allele, confirming that HOXa5 only binds this allele at rs10958403 (Figure 1E). For rs765727/ROR-α, none of the donors tested expressed a non-risk allele, preventing a similar comparison.

Taken together, these experiments indicated that the PAH signals identify enhancers active in hPAEC which target *SOX17* and contain variants likely to drive differential binding of TFs including HOXa5 and ROR-α.

### PAH-associated stimuli regulate endothelial *SOX17* expression in PAH patient cells

**Figure 2):**
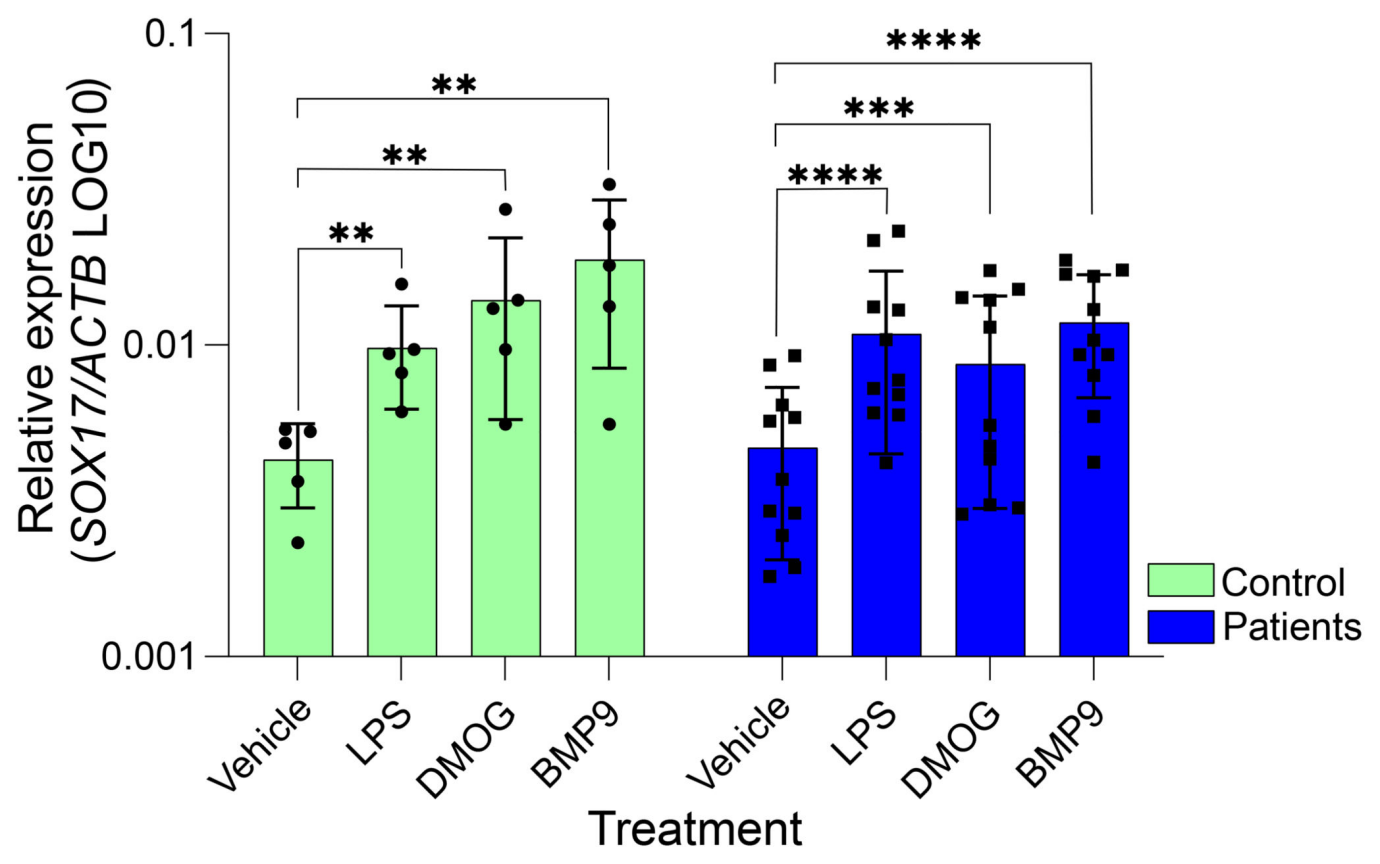
Defining the effect of upstream regulators and PAH-relevant stimuli on *SOX17*. Relative gene expression of *SOX17* compared to *ACTβ* in control and patient ECFC following exposure to known PAH stimuli. Individual data points represent individuals, n=5 controls, n=11 patients. Vehicle/treatments in 2% FBS EC media. LPS, lipopolysaccharide (2µg/ml). DMOG, Dimethyloxalylglycine (100µM), BMP9, bone morphogenetic protein-9 (10ng/ml). Ordinary 1-way ANOVA within groups compared to baseline condition with Dunnett’s multiple comparisons test. **-p<0.01, ***-p<0.005, ****-p<0.001.

To determine whether *SOX17* is regulated by factors implicated in the development of PAH, we tested *SOX17* expression in endothelial colony-forming cells (ECFC) derived from healthy controls or PAH patients after stimulation with the hypoxia mimic DMOG (dimethyloxalylglycine), the inflammatory stimulus LPS (lipopolysaccharide) and the BMPR2 ligand BMP9 (bone morphogenetic protein-9). ECFC from IPAH patients have reduced barrier function versus those from control subjects and are more susceptible to LPS-induced permeability (18). LPS treatment is not only a well-established means of stimulating permeability in pulmonary EC cultures; it also linked with selective HIF-1a stabilization (DMOG treatment in vitro) and the induction of SOX17 expression in HPMVECs (19). Expression of SOX17 has a protective effect and is required for the restoration of barrier function. ECFC derived from PAH patients with pathogenic BMPR2-variants are more susceptible to LPS-induced permeability versus control ECFC and the effect is blocked by co-treatment with BMP9 (20). Stimulation with LPS, DMOG or BMP9 significantly increased *SOX17* expression in both control (n=5) and PAH patient (n=11) ECFCs (Figure 2).

### *SOX17* regulates pathological downstream molecular pathways and functions in hPAEC

**Figure 3):**
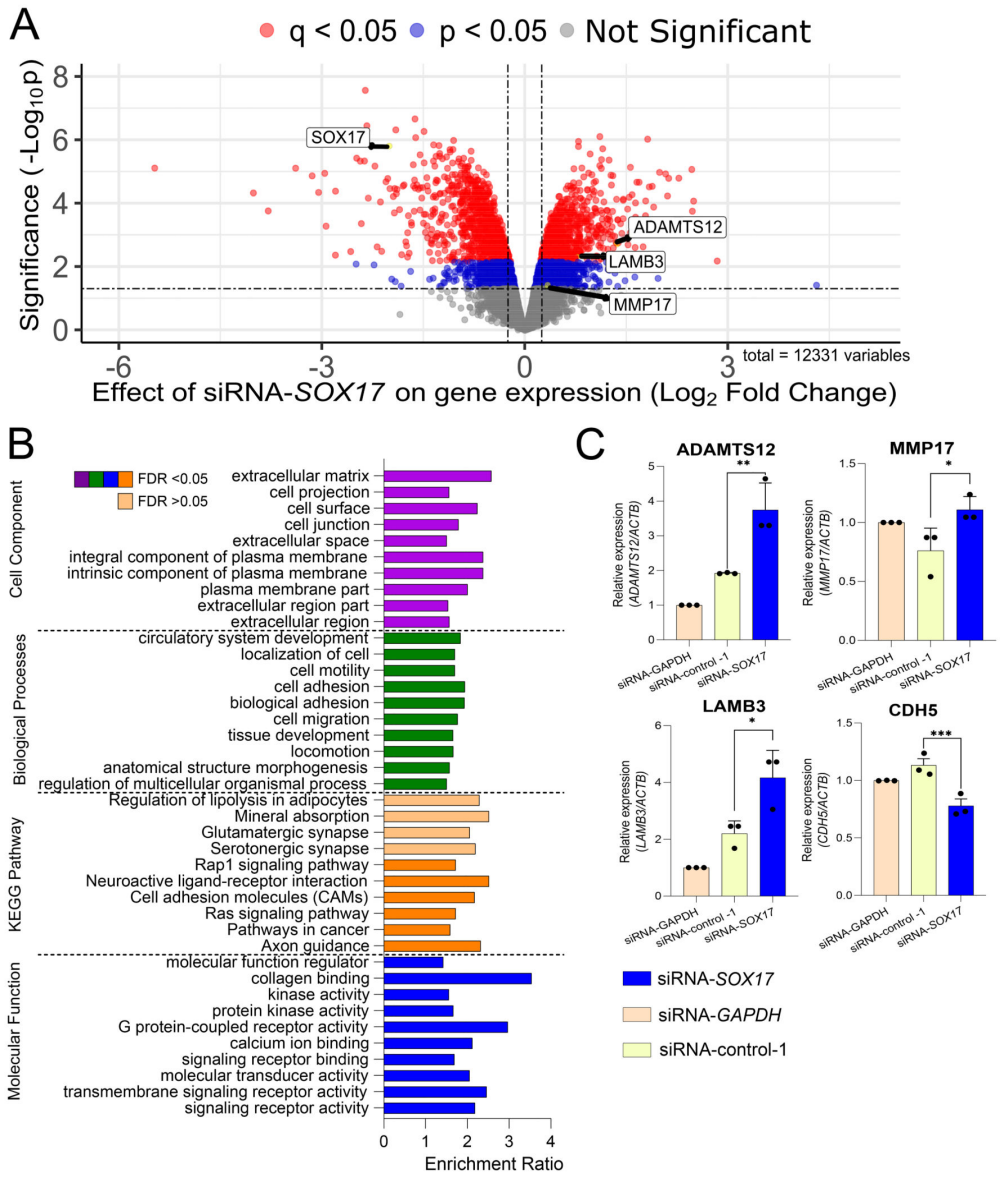
Analysis of Sox17 Manipulation in Pulmonary Vascular Cells. **A) Differentially Expressed Genes following *SOX17*-siRNA in hPAECs.** Volcano plot of the Log2 fold change (FC) between siRNA-*SOX17* and siRNA-negative control and the negative log10 p-value. Differentially expressed genes shown in blue met the cut-off points: p<0.05 and Log2 fold change <-0.25 or >0.25. Genes shown in red also met the cut-off of q<0.05. Genes of interest are highlighted in black boxes. n=4, 12196 variables. **B) Over-representation Analysis for enriched pathways and functions following *SOX17*-siRNA**. Gene ontology analysis of Cell component (purple), biological process (green), KEGG pathway (orange) and molecular function (blue) enrichment following *SOX17*-siRNA in hPAECs. Darker colours indicate f<0.05. Lighter colours indicate f>0.05. Enrichment ratios were obtained from WebGestalt. **C) Relative gene expression of gene ontology target genes by qPCR**. The change in target gene expression is normalised to *ACTβ* and all siRNA conditions are relative to the GAPDH-targeting siRNA control. Target genes are *ADAMTS12*, *MMP17*, *LAMB3, CDH5*. All statistical tests shown are paired, one-way, student’s t-test. *-p<0.05. n=3. All in hPAECs following *SOX17*-siRNA treatment for 48 hours.

The risk alleles are associated with reduced enhancer activity and therefore reduced *SOX17* expression. To determine the downstream effects of *SOX17* depletion we performed RNAseq analysis of hPAEC following modulation of *SOX17* by siRNA-mediated silencing or by CRISPR inhibition of *SOX17*-signal 1 and -signal 2 (Figure 3A, Figure S2 and S3). We identified 1717 genes that are differentially expressed following siRNA-*SOX17* knockdown (absolute log2-fold change >0.25 or <-0.25, FDR q<0.05, Figure 3A). Gene ontology (GO) shows significant enrichment of the pathways involving cell adhesion and extracellular matrix organization (ECM, Figure 3B). 451 genes were significantly down- or upregulated following CRISPR-I of *SOX17*-signal 1 and 786 genes following CRISPR-I of *SOX17*-signal 2 (absolute log2-fold change >0.25 or <-0.25, p<0.05, Figure S3). There was a significant overlap of eighty-one genes differentially expressed in both si*SOX17* and *SOX17*-signal 1 CRISPRI (p=0.0356, Supplementary Data File). Consistent with the siRNA analysis, gene ontology analysis of these differentially expressed genes shows enrichment for pathways linked to the extracellular matrix organization and cell adhesion (Figure S3). qPCR was used to validate the effect of *SOX17*-siRNA on affected genes enriched in ECM and adhesion pathways. ECM and adhesion genes *ADAMTS12, MMP17* and *LAMB3* were significantly increased when compared to a negative control siRNA, whereas *CDH5* was decreased by *SOX17* knockdown (Figure 3C). These data demonstrate that *SOX17* loss in hPAEC drives gene expression changes in pathways relevant to PAH pathology.

### Plasma proteomic differences in patients with differing *SOX17* variant genotypes

**Figure 4):**
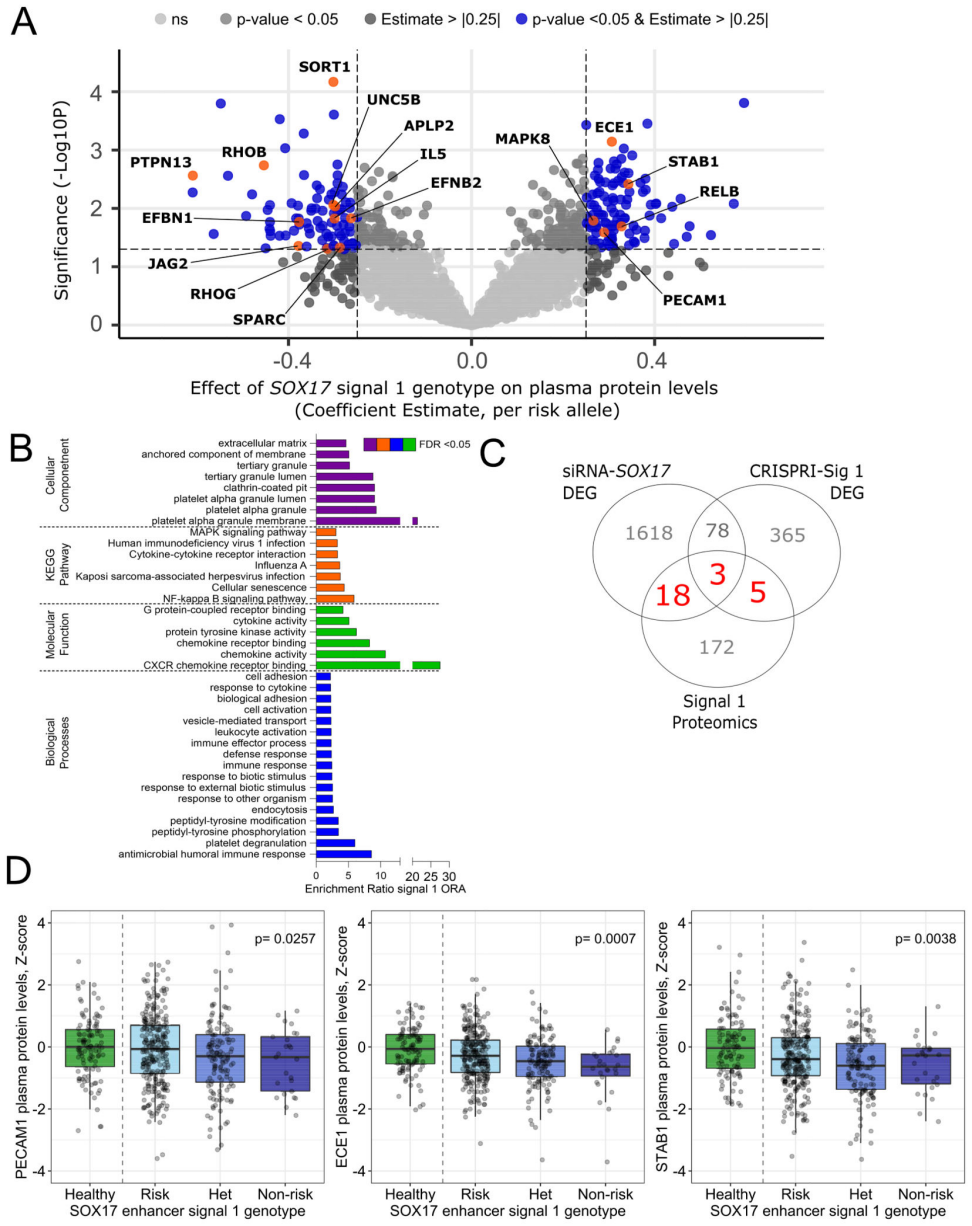
Effect of *SOX17* enhancer variant genotype on patient proteomics. **A) Linear regression for the effect of *SOX17*-signal 1 on the levels of serum proteins in patient sample.** Volcano plot of the coefficient estimate and the negative log10 p-value. Corrected for age and sex. Protein shown in blue met threshold b-estimate > |0.25| and p < 0.05. Proteins of interest are labelled and shown in orange n=431. **B) GO analysis for the significantly affected by signal 1 genotype proteins**. Gene ontology analysis of Cell component (purple), biological process (green), KEGG pathway (orange) and molecular function (blue) enrichment. Proteins met the threshold b-estimate > |0.25| and p < 0.05. Enrichment ratios were obtained from WebGestalt. **C) Comparisons of transcriptomic and proteomic analysis.** Venn diagram showing overlapping differentially expressed genes and proteins from transcriptomic and proteomic analysis from CRISPRi of *SOX17*-signal 1, siRNA-*SOX17* and *SOX17*-signal 1 enhancer variant genotype on patient proteomics. DEG, differentially expressed genes. Numbers in red show genes and proteins which are in common between all analyses. **D) Z-scored proteins in healthy controls versus patients with different genotypes in proteins of interest.** Proteins included are PECAM1, ECE1 and STAB1. Risk, homozygous for risk allele, n=271. Non-risk, homozygous for non-risk allele, n=26. Het, heterozygotes, n=134. Control, n=108.

To further examine the potential effect of common variation in the enhancer area on downstream targets of SOX17, we analysed the plasma proteome of 431 PAH patients with a known genotype of the *SOX17*-signal 1 and *SOX17*-signal 2 using linear regression analysis. We identified 198 and 161 proteins where plasma levels were significantly affected by the genotype in *SOX17*-signal 1 and *SOX17*-signal 2 SNPs, respectively (beta-estimate > |0.25| and p<0.05, Figure 4A, Figure S4). In line with our data obtained from genetic interference of *SOX17* in hPAEC, GO analysis of the plasma proteomics identified enrichment in proteins involved in regulation of adhesion and extracellular matrix for both signals (Figure 4B, Figure S4B). In addition, enrichment in proteins involved in regulation of proliferation, migration and apoptosis were found for *SOX17*-signal 2 (Figure S4). As similar processes and functions emerged from the transcriptome GO analyses, the significantly affected proteins and genes from all conditions (CRISPRI, siRNA and proteomics) were compared in detail (Figure 4C, Figure S4C). Six proteins were affected by patient genotypes at both *SOX17* signals (IL5, PTPN13, STAB1, SUGT1, GAPDH and ADGRG5). There were 26 genes in common between *SOX17*-signal 1 proteomics and CRISPRI or siRNA analyses (Figure 4A, Supplementary Data File) and 23 genes between *SOX17*-signal 2 proteomics and CRISPRI or siRNA analysis (Figure S4). These included Secreted Protein Acidic And Cysteine-Rich (SPARC), Platelet And Endothelial Cell Adhesion Molecule-1 (PECAM1), Endothelin Converting Enzyme-1 (ECE1), Collagen Type XVIII Alpha-1 (COL18A1), Interleukin-5 (IL5) and Stabilin-1 (STAB1) which have been previously associated with PAH (21)(22)(23)(24) (Figure 4D, Figure S4). These analyses suggest that differences in *SOX17* enhancer activity associated with PAH risk alleles lead to changes in the plasma proteome with pathologically relevant functions.

### Functional impact of loss of SOX17 in cultured hPAEC

**Figure 5):**
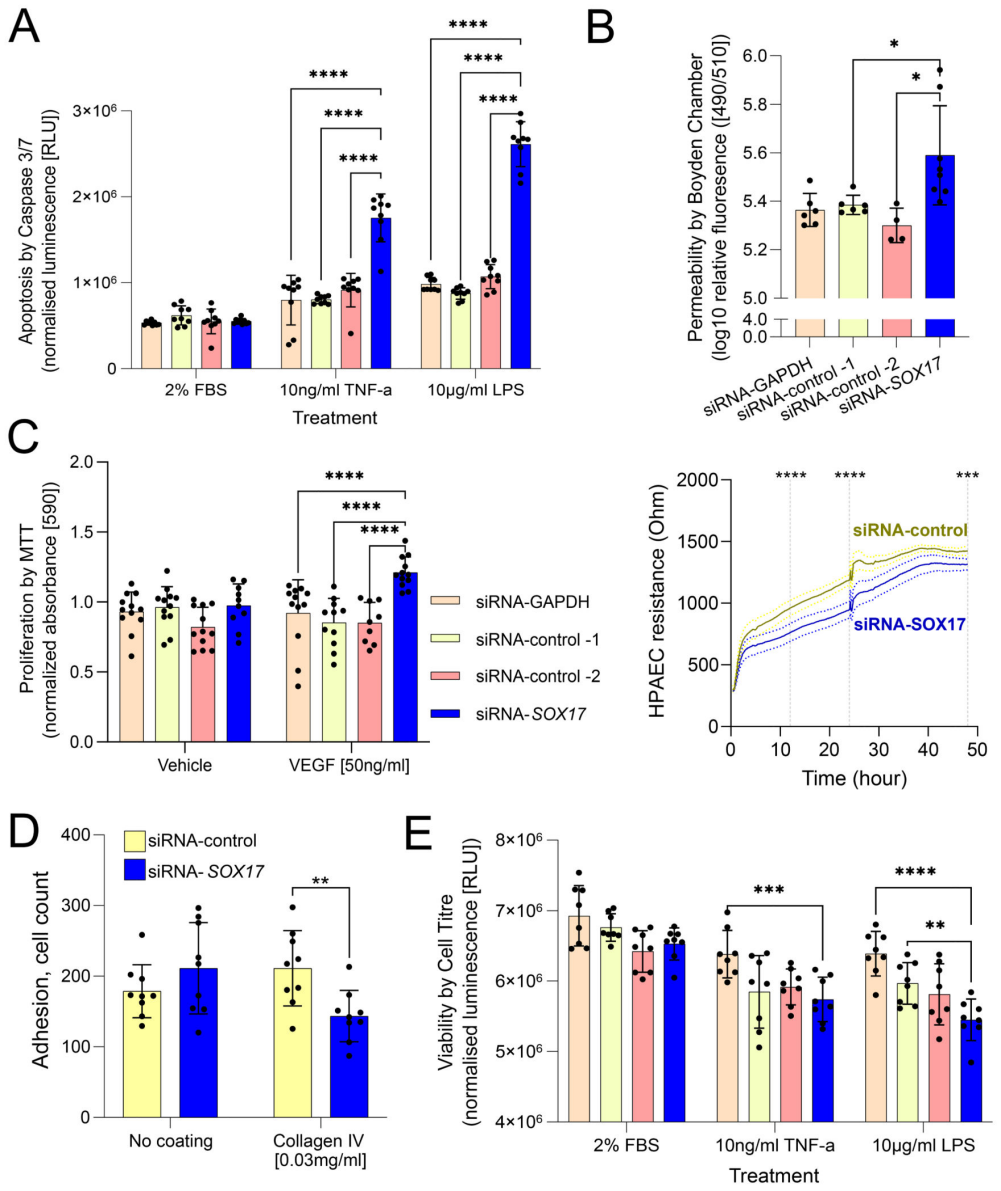
Functional Analysis of Sox17 loss in hPAEC. All following siRNA treatment for 48hrs with scrambled controls, targeting *SOX17* or unrelated gene *GAPDH*, with relevant stimuli as indicated. **A) Caspase 3/7 apoptosis assay.** 2% FBS was used as a proliferation control. TNFα and LPS were used as pro-apoptotic inflammatory stimuli. **B) Permeability barrier function assays. Upper, Boyden chamber FITC dextran. Lower, Electrical cell Substrate Impedance Sensing (ECIS) measurements at 0-48h. C) MTT proliferation assay.** Vehicle, 0.1% BSA in PBS. Vascular endothelial growth factor (VEGF) was used as a pro-proliferative stimulus. **D) Adhesion cell counting assay.** Adhesion was compared between cells in wells with no coating or pre-coated with collagen IV. **E) Cell Titre viability assay.** Performed under same conditions as caspase assay. TNFα, tumour necrosis factor-α; LPS, lipopolysaccharide. Statistical tests used in A, C and D are ordinary two-way ANOVA with Dunnett’s multiple comparisons test. The statistical test used in B is an ordinary one-way ANOVA with Dunnett’s multiple comparison test. *-p<0.05. **-p<0.01. ***-p<0.005. ****-p<0.001. Minimum n=3 experiments for all, culture replicates plotted.

To determine the functional impact of loss of *SOX17*, we exposed hPAEC to relevant stimuli following siRNA-mediated *SOX17* knockdown. SOX17 knockdown increased hPAEC apoptosis (caspase-3/7 activity) in response to either TNF-α or LPS as compared to siRNA controls (p<0.001, Figure 5A), with hPAEC viability being either unchanged or decreased with siRNA-*SOX17* when compared to siRNA controls (Figure 5E). Knockdown of *SOX17* led to an increase in hPAEC monolayer permeability, as measured by a transwell assay (both p<0.05, Figure 5B) and by electrical impedance assays (p<0.001, Figure 5B lower panel). Adhesion to collagen IV was significantly decreased in *SOX17*-depleted hPAECs compared to controls (Figure 5D), while SOX17 knockdown increased VEGF-induced hPAEC proliferation as determined by MTT assays (p<0.001, Figure 5C). These results suggest that *SOX17* loss in hPAEC fundamentally changes their function mirroring changes observed in patient PAEC.

### Connectivity Map prediction of rescue compounds for drug repurposing

**Figure 6):**
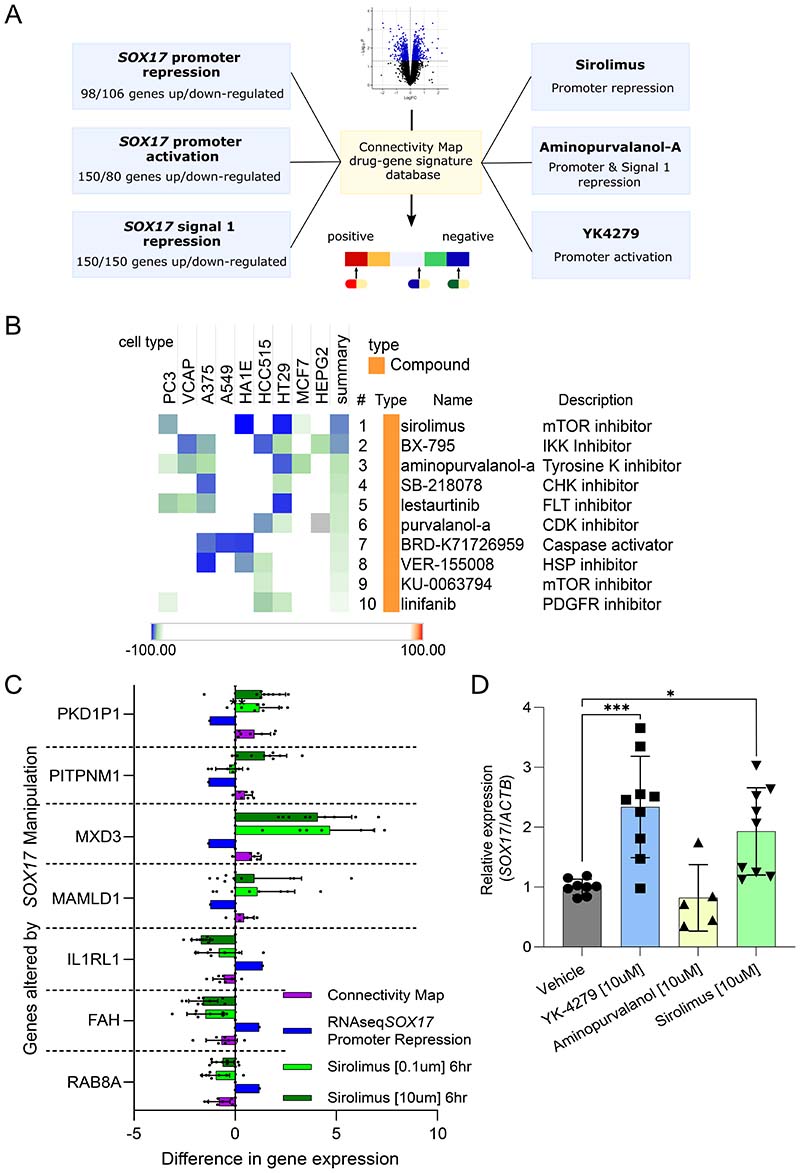
Repurposing of Compounds to Rescue loss of *SOX17* Function in PAH. **A) Summary diagram showing the prediction of compounds from omics signatures using connectivity map (CMap) database signatures of drug or gene manipulation in reference cell lines.** Omics signatures used to query the Cmap database are shown on the left. Predicted compounds are shown on the right. **B) Cmap analysis results for *SOX17* promoter repression using CRISPR-inhibition in human pulmonary artery endothelial cells (hPAEC).** Cell types are shown. Tau scores within heatmap indicate percentage of all possible compounds in CMAP and cell lines tested the specific result is more connected than. Sirolimus overall is negatively connected more strongly than other compounds, with a summary Tau of -96.94. **C) Relative gene expression of target genes in the sirolimus perturbagen signature.** Target genes were *PKD1P1, PITPNM1, MXD3, MAMLD1, IL1RL1, FAH* and *RAB8A*. Connectivity map, perturbagen z-score taken from the Cmap. RNAseq *SOX17* promoter repression via CRISPRI (n=3), fold change from RNAseq analysis of DEG following CRISPRI of the *SOX17* promoter (n=3). Sirolimus [0.1μm/10μm] (n=3), the change in target gene expression is normalised to *ACTβ* and all siRNA conditions are relative to the GAPDH-targeting siRNA control in hPAECs following sirolimus exposure at the stated concentrations. **D) Relative expression of *SOX17* following CMap compound exposure by qPCR in hPAEC.** The change in *SOX17* expression is normalised to *ACTβ* and all compounds are relative to the vehicle control. Ordinary 1-way ANOVA of conditions compared to vehicle with Dunnett’s multiple comparisons test. *-p<0.05, ***-p<0.005. n=3.

To predict if available drug compounds could be repurposed to reverse the gene changes associated with *SOX17* dysfunction, we interrogated the CMap database. The CMap contains transcriptomic signatures of thousands of compounds' effects on multiple cell lines, allowing comparison of user-generated signatures (Figure 6A). To test effects most relevant to the common and rare *SOX17* variants associated with PAH, we generated *SOX17* hPAEC signatures comprised of differentially expressed genes following CRISPR-inhibition of *SOX17*-signal 1 or the *SOX17* promoter, or CRISPR-activation of the *SOX17* promoter. *In-silico* analysis of these signatures show the compounds sirolimus, aminopurvalanol-a and YK4279 to match our *SOX17* signature in hPAEC (Figure 6B). Sirolimus and aminopurvalanol-a are predicted to reverse (negatively connected to) *SOX17* promoter repression (Tau score:-96.94 and -95.17 respectively). Aminopurvalanol-a is also negatively connected to *SOX17*-signal 1 repression (-99.65). YK4279 is predicted to mimic *SOX17* promoter activation (positive connection, +93.88). Comparisons of each compound’s signature from the Cmap and our RNAseq signature resulted in the gene lists shown in Table S3. For each compound, we selected a panel of genes that showed consistent directional changes across multiple cell lines in the Cmap and tested their expression by qPCR in hPAEC following compound exposure. For sirolimus, all tested genes showed the predicted expression change (Figure 6C). For aminopurvalanol-a, only half of the genes tested changed in the direction predicted by the Cmap signature (Supplementary Figure 5). For YK4279, all the gene expression changes tested were as predicted (Supplementary Figure 5). To determine whether the changes seen could be directly through *SOX17* effects we measured *SOX17* levels in the treated hPAEC and found that both YK-4279 and sirolimus significantly increased *SOX17* relative expression compared to vehicle (Figure 6D). These data confirmed that Cmap-predicted compounds can successfully reverse some genetic changes associated with *SOX17* dysfunction in hPAEC.

### Animal knockout of *SOX17* enhancer worsens PH

**Figure 7):**
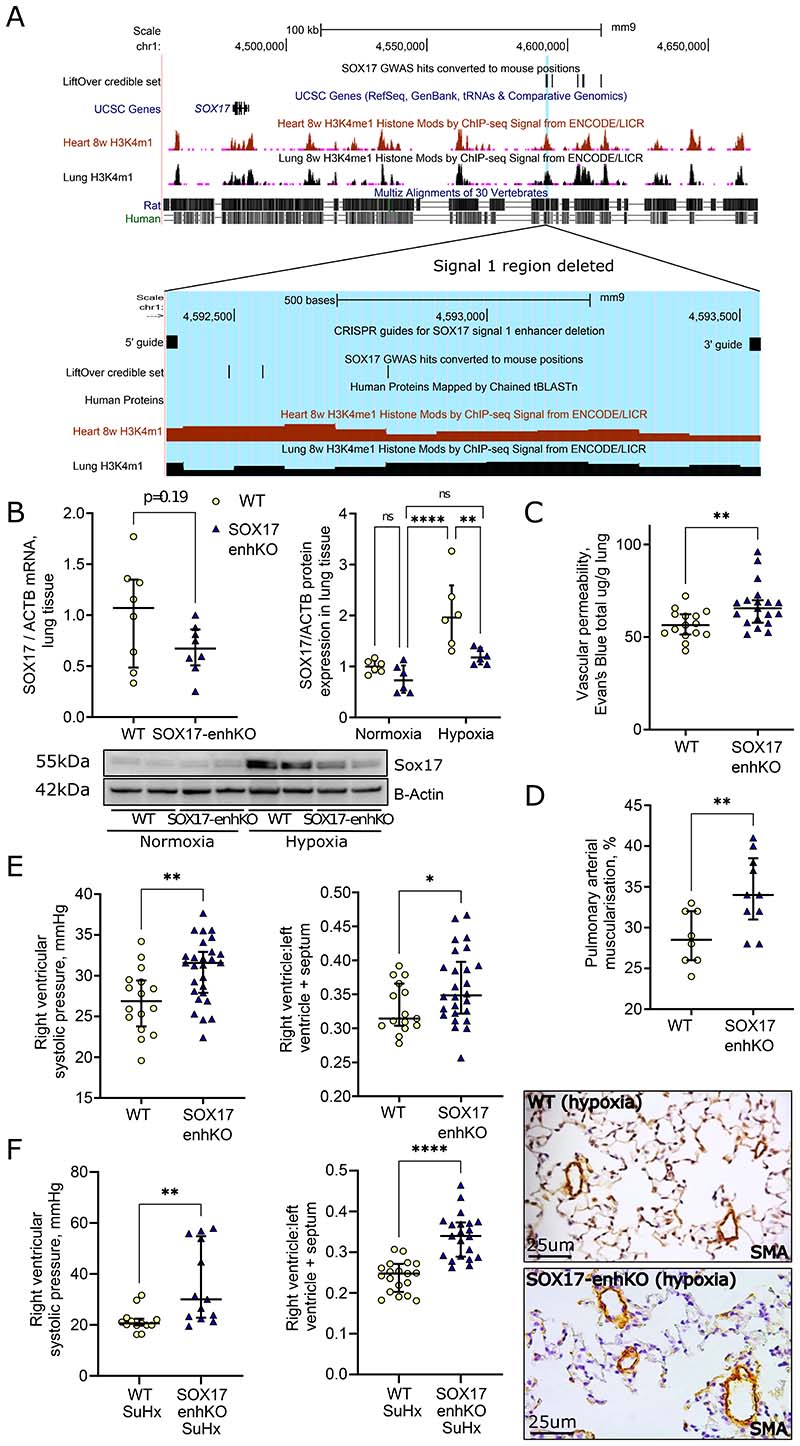
*SOX17*-signal 1 enhancer knockout mice develop more severe PH in hypoxia. **A) Map of PAH *SOX17* enhancers in mouse genome.** Black bars labelled ‘Enhancer and GWAS hits LiftOver’ and ‘User track’ indicate conserved genomic regions from the human *SOX17* enhancer peaks. Lines indicate positions of variants associated with PAH in the human GWAS. Mouse epigenomic H3K4m1 data show that this area is also likely to be an active regulatory region in mice. Blue region highlights enhancer targeted for deletion. **B)** ***SOX17* mRNA and protein expression**. Taken from lung tissue following 3 weeks hypoxia and compared to beta-actin (ACTB) housekeeping gene/protein. *-p<0.05, **-p<0.01 versus WT (unpaired t-tests). n=6 and n=8. **C)** **Lung vascular permeability.** Determined by Evan’s blue dye in wildtype (WT) and *SOX17* enhancer knockout lung tissue following 1 week of hypoxia. *-p<0.05, **-p<0.01 versus WT (unpaired t-tests). n=15 and n=18. **D)** **Pulmonary vascular muscularisation**. Determined from smooth muscle actin (SMA) and Elastic Van Gieson (EVG) staining. *-p<0.05, **-p<0.01 versus WT (unpaired t-tests). n=7 and n=10. **E)** **Right ventricular systolic pressure (RVSP)** and **RV hypertrophy (RVH)** indices of PH severity in WT and SOX17 enhancer knockout mice following chronic hypoxia (10% O2 3 weeks). *-p<0.05, **-p<0.01 versus WT (unpaired t-tests). **F) Right ventricular systolic pressure (RVSP)** and **RV hypertrophy (RVH)** indices of PH severity in WT and SOX17 enhancer knockout mice following SUGEN 5 mg/kg and 12% O2 for 3 weeks (SuHx). **-p<0.01, ****-p<0.001 versus WT SuHx (unpaired t-tests).

We have successfully generated mice lacking the 747bp enhancer region containing *SOX17* GWAS signal 1 using CRISPR/Cas9-mediated deletion on C57BL/6 background (Figure 7A, Figure S6). The SOX17-enhKO and wild type (WT) mice kept in normoxia did not show significant differences in right ventricular systolic pressure (RVSP) and right ventricular hypertrophy index (RVH, RV/LV+septum, Supplementary Figure 6B). We then exposed the mice to hypoxia (normobaric, 10% oxygen) for 1 and 3 weeks. SOX17 protein levels in the lungs of the SOX17-enhKO mouse were reduced in comparison to WT when exposed to hypoxia (Figure 7B). After 1-week hypoxia exposure SOX17-enhKO mice demonstrated significantly increased lung vascular permeability in comparison to WT (Figure 7C). At 3 weeks, chronic hypoxia-induced PH severity was intensified in the SOX17-enhKO animals as demonstrated by increased peripheral pulmonary vessel muscularization (Figure 7D), RVSP (Mean+/-Std.Deviation: 26.93+/-3.916 vs. 30.66+/-3.856 mmHg, p=0.004), RVH and RV/body weight (Figure 7E, Supplementary Figure 6C).

To confirm these findings using a different model of PH, the same SOX17-enhKO were studied under different levels of hypoxia and SUGEN (SU-5416). Using the standard protocol of 20 mg of SUGEN and 3 weeks of hypoxia, there were no significant differences in the severity of PH that developed between, SOX17-enhKO and WT mice. However, SOX17-enKO were more susceptible to developing SUGEN/hypoxia-PH as evidenced by the development of severe PH at lower levels of SUGEN-hypoxia (5 mg/kg and 12% O2) which did not produce PH in WT littermates (RVSP: 21.66+/-4.43 vs 35.94+/-15.36 mmHg p=0.006, Figure 7F and Supplementary Figure 7). Thus, in two independent laboratories using different PH models it was shown that *SOX17* enhancer knockout increases susceptibility to and severity of PH.

## Discussion

**Figure 8):**
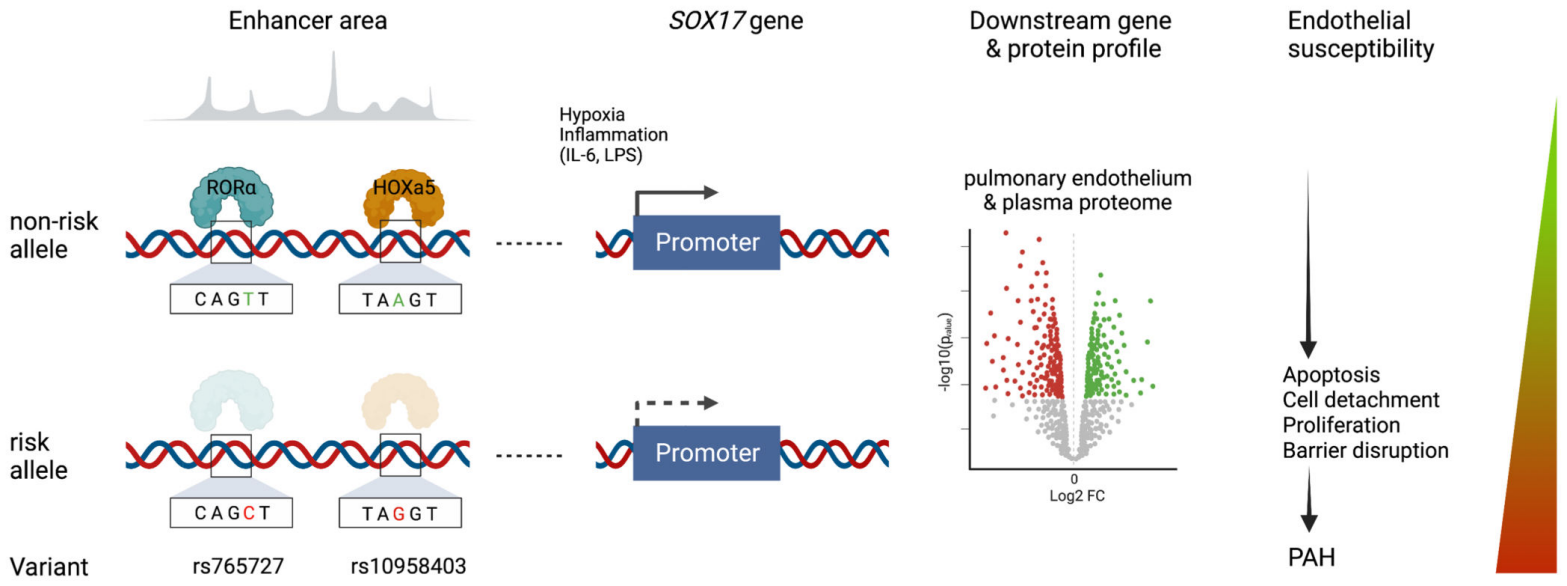
Summary figure. Schematic depicting overall study findings from identification of RORα and HOXa5 as transcription factors binding PAH-associated variants in enhancers upstream of *SOX17,* through regulation of *SOX17* by PAH stimuli, downstream effects of *SOX17* on gene and protein expression profiles and endothelial cell behaviour, culminating in worsened PAH in *SOX17*-enhancer knockout mice.

Here we provide novel insight into how two independent common genetic variants upstream of the key endothelial transcription factor, *SOX17,* can increase susceptibility to PH (Figure 8). In brief, variation at rs765727 and rs10958403 in putative *SOX17* enhancer signals 1 and 2 determine the binding of two transcription factors, RORα and HOXA5, respectively. Allele-specific reduced binding of either factor leads to reduced *SOX17* expression. SOX17 is crucial for maintaining endothelial cell homeostasis and its loss drives abnormal proliferation, apoptosis and adhesion, and impairs endothelial barrier integrity. Our prediction that this would increase susceptibility to PH was confirmed in mice lacking *SOX17*-signal 1 enhancer signaling exposed to hypoxia with and without Sugen.

Defining the importance and biological function of GWAS signals in complex diseases is a challenge. Many are located in non-coding regions of the genome which complicates interpretation. Confirmed examples of variation in enhancer regions causing disease are few, including BCL11 in sickle cell disease (25) and FTO in obesity (26). *SOX17*-signal 1 and -signal 2 are located inside a topologically associated domain (TAD) in which *SOX17* is the only gene, making it the most likely target for these two enhancers (7). A significant and specific decrease in *SOX17* expression was observed following CRISPR-inhibition of *SOX17*-signal 2 and CRISPR-deletion of *SOX17*-signal 1, establishing that these genomic signals are associated with the regulation of *SOX17* expression. EMSA (and ChIP-qPCR for HOXa5) demonstrated that HOXa5 or ROR-alpha bind to the non-risk alleles present at *SOX17*-signal 1 and signal 2 respectively. Thus, an individual homozygous for one or both risk alleles would be more resistant to HOXa5 and/or ROR-alpha induced SOX17 expression than an individual hosting non-risk alleles. While it is not a requirement that these transcription factors are themselves associated with PAH, HOX transcription factors expression have been shown to differ in PAH lung tissue (27) which may further exacerbate the effect of the differential binding affected by the disease-driving variant.

*SOX17* has established roles in systemic artery endothelial cells, but little is known about its role in the pulmonary vasculature. Establishing the downstream targets of *SOX17* in hPAEC is vital to understanding how it mediates the risk of developing PAH. Our transcriptomic analysis identified several specific candidates and enrichment of gene pathways implicating dysregulation of endothelial functions and extracellular matrix (ECM) associated with loss of *SOX17*. This was supported by our plasma proteomic analysis between patients with risk or non-risk *SOX17* enhancer genotypes, where we found an enrichment of adhesion- and ECM-associated proteins. The basement membrane is thicker in the lungs of iPAH patients, and regulation of the ECM is established as an important factor in PAH (28). We have demonstrated here that the ECM-linked genes *LAMB3, ADAMTS12* and *MMP17* were affected at the mRNA level by *SOX17* knockdown in independent experiments. *ADAMTS12* and *MMP17* expression increased following a loss of *SOX17*, suggesting they may be important contributors to the functional effects of *SOX17* loss in hPAEC. *ADAMTS12* is a disintegrin and matrix metalloproteinase gene with an important role in ECM composition (29). Coupled with *MMP17*, it is part of a large family of matrix metalloproteinase genes which may have important roles in pathophysiological functions in hPAEC in PAH (30). Although *MMP17* has not been directly linked to PAH, loss of function variants, found in familial studies, confer a greater risk of aortic aneurysm in mice through dysfunctional ECM filament deposition and an enlarged aortic lumen (31).

Analysis of the effect of patient *SOX17* enhancer genotypes at *SOX17*-signal 1 and signal 2 on plasma protein levels identified a large number of significantly affected proteins enriched in the regulation of adhesion and extracellular matrix, proliferation, migration and apoptosis, all processes crucial for the development of PAH. There were proteins and genes that were significantly affected both by the *SOX17*-signal 1 and signal 2 patient genotypes and by the loss of *SOX17* expression via siRNA or CRISPRi in hPAEC, suggesting circulating proteins might reflect *SOX17* dysfunction in PAH. Interesting candidates among these are SPARC, which was found at increased levels in lung of IPAH patients and is involved in the regulation of PASMC proliferation (21), ECE1, which is involved in the cleavage/transformation of Endothelin 1 to its active form (22), PECAM-1, which plays a role in the adaptation of endothelium to shear stress (23) and COL18A1, which when cleaved produces Endostatin, a protein whose serum levels are correlated with disease severity and survival in PAH (24).

That rare pathogenic variants in *SOX17* also drive PAH development and are associated with more severe PAH and younger age (32), emphasises the importance of this gene and related pathways as a therapeutic target. To pursue this, we explored CMap for novel candidates that might rescue *SOX17* activity. Three candidate compounds emerged from our screen using hPAECs, all suitable for exploratory studies in humans. Sirolimus is a specific inhibitor of mTOR and an allosteric inhibitor of mTORC1. Another immunosuppressor, tacrolimus, has entered clinical trials in PAH. Tacrolimus is a calcineurin inhibitor but was selected for study at low doses expected to modulate *BMPR2* signalling based on a screening strategy and hence has a distinct mechanism to sirolimus. Tacrolimus was found to be well tolerated and to improve 6-min walk distance and echocardiographic parameters of heart failure in PAH patients in a phase IIa safety and tolerability study. Although the small number of patients studied did not result in statistical significance, the findings did support the study of tracrolimus in a phase IIb efficacy trial (33). Several prior studies have shown the efficacy of sirolimus in reversing animal models of PH (34) and an albumin-bound nanoparticle form of sirolimus is currently undergoing a phase I/II clinical trial and has so far shown no safety concerns and an early efficacy signal (35). It would be of interest to gauge the importance of regulation of *SOX17* and its signalling in the efficacy of sirolimus and addition of biomarker measurements (e.g. SPARC) in future trials would be valuable.

Aminopurvalanol-a (also known as purvalanol-a) is a CDK1/cyclin B inhibitor that arrests proliferating cells in the G2/M stage of the cell cycle and prevents proliferation. It has been investigated in human microvascular endothelial cells as an anti-angiogenic drug and was shown to inhibit proliferation, increase apoptosis and prevent tube formation (36). Some CDKs are upregulated following *SOX17* silencing in arterial endothelium (37). In both monocrotaline and Sugen/hypoxia PAH rat models, the CDK inhibitor palbociclib reversed PAH pathology including right heart hypertrophy and pulmonary remodelling (38). Both CDK inhibitors dinaciclib (inhibits CDK1, 2, 5 and 9) and palbociclib (inhibits CDK4 and 6) reduced proliferation in SMCs (38).

A third compound from the Cmap screen, YK-4279, is an ETS family inhibitor that has undergone pre-clinical efficiency trials as an anti-lymphoma drug (39). ETS family members are known oncogenes whose aberrant expression is found in many solid tumours (40). They also have roles in vascular development and maintenance (for full review: (41)). YK-4279 specifically inhibits ERG transcription and ERG-mediated cell migration and proliferation in prostate cancer (42). The ERG TF is essential for EC homeostasis and has recently been shown to bind to a super-enhancer upstream of the *SOX17* gene in HUVEC, suggesting a possible role for ERG in the regulation of *SOX17* in this cell type (43).

*SOX17* knockdown in mice results in embryonic lethality with heart defects and enlarged veins (44). Endothelial-specific knockdown in either embryonic or adult mice causes defects in artery specification and in-utero lethality (10,45). SOX17 endothelial-(Cdh-CreER)-knockout exacerbated hypoxic PH which was sustained despite return to normoxia for 3 weeks. Consistent with our findings in si-SOX17-treated human PAEC, hyperproliferation of ECs was prominent in Sox17knockout/hypoxic mice by Ki67 staining (46). These studies support a role for *SOX17* in arterial endothelial cells *in-vivo*. We can report that mice lacking *SOX17*-signal 1 are viable but show increased susceptibility to hypoxia or combined SUGEN-hypoxia associated PH. Consistent with our in vitro data and prior reports in other vascular beds (47,48), the *SOX17* enhancer knockout mice exhibited elevated vascular permeability compared to WT animals. This illustrates the importance of fine tuning *SOX17* levels to modulate endothelial barrier function under pathological conditions. Whilst there was no phenotype apparent in the mice under normoxic conditions or more severe SUGEN-hypoxia, it is remarkable that deletion of an enhancer alone, rather than a complete or partial gene deletion (as required for *BMPR2*) was sufficient to lead to a worse PH phenotype in two independent laboratories, under hypoxia or lower levels of SUGEN-hypoxia than drove PH in wildtype animals. The concept that a ‘second hit’ may be required to exhibit the PH phenotype is well understood and in patients harbouring a *SOX17* enhancer risk allele, this may comprise inflammation or drug toxicity as well as hypoxia.

It remains possible that other transcription factors also bind differentially to PAH-associated *SOX17* enhancer variants. In our EMSA experiments, use of a ROR-α antibody lead to loss of signal rather than a clear supershift, which is most likely due to the antibody preventing formation of the ROR-α-probe complex. SOX17 is not included on the proteomics platform used in this study. The numbers of homozygotes for rarer variant alleles are small so variability in these measurements is high. Validation of key findings in hPAECs with (naturally occurring or knocked-in) variants in *SOX17*-signal 1 or 2 in addition to the complete deletion of the enhancer would have even more robustly supported functional conclusions. The effects would likely be more subtle than deletion or inhibition of the enhancer requiring larger n numbers. Aminopurvalanol did not affect SOX17 expression but was identified as a drug which can alter the downstream transcriptomic signature produced by SOX17 loss.

In summary, we provide comprehensive insight into how common variation influences the binding of HOXa5 and RORα to enhancers upstream of *SOX17* and can reduce susceptibility to PH. Loss of *SOX17* leads to downstream alterations in extracellular matrix regulation and hPAEC function. *SOX17* is a priority for therapeutic rescue and predicted compounds which restore endothelial gene expression offer candidates for future investigation.

## Supplementary Material

Supplementary Material

Unedited gels

## Clinical Perspective

### 1) What is new?

- *SOX17* enhancer common variants associated with PAH development alter binding of transcription factors and thereby levels of SOX17.
- Loss of *SOX17* enhancer region drives worsening of PH in animal models.
- Gene and protein signatures driven by SOX17 dysfunction are identified and potential rescue therapeutic candidates proposed.

### 2) What are the clinical implications?

- Higher confidence in the causal variants of common genetic risk of PAH is provided.
- This research describes the biological pathways that are likely affected in PAH patients carrying risk genotypes of SOX17, thereby improving our understanding of the pathogenesis of this deadly condition.
- Compounds targeting the pathways to restore physiological SOX17 signaling in PAH patients are a priority for further investigation.
