# Supplementary material for: SOX17 enhancer variants disrupt transcription factor binding and enhancer inactivity drives pulmonary hypertension
Source: Circulation. Author manuscript; Available in PMC 2023 May 25. (PMC7614572; doi:10.1161/CIRCULATIONAHA.122.061940)
Supplement: Supplementary Material [file EMS173250-supplement-Supplementary_Material.pdf]

Supplemental Materials

Supplemental Methods

Cell Culture

HEK293FT (Invitrogen, US) and hPAECs (PromoCell GmbH, Germany and Lonza, USA) were cultured at 37°C in 5% CO2. HEK293FTs were cultured in Dulbecco's Modified Eagle Medium (DMEM, Sigma Aldrich) supplemented with 10% fetal bovine serum (FBS, Sigma Aldrich), 1% L−Glutamate, 1% Sodium pyruvate and 1% essential amino acids (All Sigma Aldrich). Fibronectin (Sigma Aldrich) was used as a seeding agent unless stated otherwise for hPAECs which were cultured in 10% FBS endothelial growth medium 2 (EGM2, (PromoCell or Lonza)), with additional supplements provided by the manufacturer.

Primer and Probe Design

All primers and probes (Life Technologies Limited, UK) were resuspended according to the manufacturer's instructions. Biotin-labelled and 'cold' (unlabelled) probes for EMSA (Table S4) were designed based on the 42 base pairs centred on rs10958403 or rs765727 with either the non-risk (A/C) or risk (G/C) allele as the 21st base. Competitor probes to investigate transcription factor binding to the target probes were designed based on replacing the 6/8 base pairs at the centre with the consensus sequence of the transcription factor. CRISPR−INH guides (Table S1) were designed using Tefor-CRISPOR software (version 4.7). Primers for use in qPCR (Table S5) were designed using PrimerBlast (NCBI, USA).

CRISPR-Manipulation

To inhibit *SOX17*-signal 2 and -signal 1, CRISPR−Inhibition (CRISPR-I) was used. Guides (Table S1) were cloned into a lentiCRISPRi vector coupled with a KRAB inhibitor (Lenti-KRAB, Addgene #118154) or a SAM enhancing element (Lenti-SAM, Addgene #89567), transformed into Stbl3 E. coli and selected on ampicillin positive LB-agar (Sigma Aldrich) plates overnight. Guide RNAs targeting blue fluorescent protein (BFP) (Addgene, #118157), green fluorescent protein (GFP) (Addgene #118158) or the promoter of SOX17 were also cloned into the lentiCRISPRi vector (Addgene #11815) and used as controls in each experiment.

To delete SOX17-signal 1, CRISPR-Deletion (CRISPR-D) was used. Two guides (Table S1) against SOX17-signal 1 were cloned into a pSpCas9(BB)-T2A-HygR vector (#118153) using a one-step dual CRISPR/Cas9 guide RNA cloning protocol (49). In short, two guide RNAs were ligated to the pScaffold-H1 (#118152), which was subsequently cloned into pSpCas9(BB)-T2A-HygR using the BsmBI cloning sites. The pSpCas9(BB)-T2A-HygR vector containing gRNA or empty vector were electroporated in HPAECs using the Amaxa electroporation system. Transfected HPAECs were selected with two rounds of 33µg/ml hygromycin. Subsequently, genomic DNA and RNA was isolated with designated kits (Qiagen). Polymerase chain reaction was performed to confirm knockout of the designated enhancer region. After generation of cDNA using reverse transcriptase, quantitative PCR was performed to evaluate the effect of the enhancer knockout on SOX17 expression.

Plasmid DNA was extracted using the GenElute Plasmid MiniPrep Kit (Sigma Aldrich). After sequence verification using Genewiz (Leipzig, Germany) universal U6 primer, colonies containing lentiCRISPRi vector were used for Pureyield Plasmid Midiprep (Promega, USA). Co-transfection into 70% confluent HEK293FT cells was performed with PEIpro transfection reagent (Polyplus Transfection, France). The purified plasmids were transfected with lentiviral packaging plasmids pMDLg/pRRe, pRSV-Rev and pMD2.G (Addgene #12251, #12253 and #12259). 96 hours after transfection, lentiviral particles were

harvested using Lenti-X concentrator (Clontech, USA). hPAECs were transduced with 10 µl of concentrated lentiviral particles and, after 48 hours, antibiotic selection was performed with blasticidin [10 µg/ml] (ThermoFisher) for 72 hours. Following this, the blasticidin was removed and EGM2 was added for a minimum of 2 hours before continuing onto mRNA extraction using the Rneasy Plus kit (Qiagen, UK).

EMSA & Supershift

To investigate transcription factors (TF) whose binding may be affected due to the *SOX17* variants, in−silico analysis was used. The CIS−BP database (Build 1.02. Dec 2018), PROMO (TransFac, version 8.3. Dec 2018) and ConSite (Dec 2018) were used to assess differential binding of TFs to a sequence containing the risk (G/C) or non-risk (A/T) allele. Those with differential binding were assessed for expression in cultured hPAECs using RNAseq data from hPAECs. Only those with detectable expression in hPAECs were considered for further investigations. Alternative public databases (GDS2565 for HPAEC transcriptomics, the Betsholtzlab for single cell sequencing in mouse adult lung and Cellxgene for single cell sequencing in human adult lung) were also considered for candidates before final selection for experiments (https://betsholtzlab.org/VascularSingleCells/database.html and https://cellxgene.cziscience.com/e/krasnow_lab_human_lung_cell_atlas_10x-1-remixed.cxg/).

To investigate the differential binding of TFs to the risk and non-risk allele present at the *SOX17* variants, an EMSA was performed. Nucleic proteins were extracted from hPAECs using the NE-PER Nuclear and Cytoplasmic Extraction Reagents kit (ThermoScientific). Proteins were concentrated using an Amicon centrifugal filter (300 Kd NMWL, MERCK, Germany) and protein concentration was assessed using a Pierce Coomassie (Bradford) protein assay kit (ThermoScientific).

Biotin labelled probes, un-labelled competition (cold) probes and transcription factor competition (cold) probes (Table A) were annealed with 1x annealing buffer (10x: TRIS [100 mM], EDTA [10 mM], NaCl [500 mM]. All Sigma Aldrich) by heating for 5 minutes at 95°C followed by gentle cooling to room temperature for two hours. Competition EMSA were performed using the Light-Shift Chemiluminescent EMSA protocol (ThermoScientific) with both the risk bio-probe and the non-risk bio-probe and their corresponding specific and non-specific competition probes. To further assess the binding of specific transcription factors to the target sequences, a TF competition EMSA was used. Transcription factor competition cold probes (Table S4) were designed based on replacing the 6/8 base pairs at the centre of the unlabelled probe with the consensus binding sequence of the transcription factor in question. The consensus binding sequences of these TFs were found through mining the publicly available database JASPAR (jaspar.genereg.net).

To investigate which transcription factor binds to the *SOX17* variants, a supershift assay was used. The methods, materials and reagents discussed previously were utilised with the addition of a RAR-related orphan receptor alpha (ROR-alpha) antibody (sc-518081 X, Santa Cruz, USA) or rabbit IgG (sc-2025, Santa Cruz).

ChIP qPCR

Confluent HPAECs (n = 4 donors) were washed with PBS, scraped and transferred to Eppendorf tubes. Paraformaldehyde to a final concentration of 1% to crosslink histones, followed by three PBS washing steps. Cells were lysed using L1 cell lysis buffer with protein inhibitor cocktail followed by L2 nuclear lysis buffer with protease inhibitor cocktail. DNA was sheared to a length of 200-1000 base pairs and precleared with Protein G beads. For each ChIP reaction 100uL sample was diluted in 400uL dilution buffer with 1% bovine serum albumin. Non-specific IgG control antibody (2.5ug), anti-HOXa5 (2.5ug, clone C-11 SC-365784) or anti-RORa (2.5ug, clone C-7 SC-518081) were added for

immunoprecipitation (4oC overnight). Protein G magnetic beads were added, followed by washing steps including low salt buffer, high salt buffer, LiCl wash buffer and three times Tris/EDTA buffer. DNA was eluted in 100ul elution buffer. Crosslinking was reversed by adding 3M NaCl and proteinase K and incubation at 55oC (1h), 65oC (4h) and 95oC (10min). DNA was purified using the Qiagen DNA isolation kit. A polymerase chain reaction was performed on the ChIP sample with primers for the regions containing SNP rs10958403 (TAGAA GCGAC GCTGC ATGT; rs10958403 Forward; GGGAG GCCTT TTGTA TGGCA; rs10958403 Reverse) or rs765727 (CTGGA TGGTT TCCTG TGGGT; rs765727 Forward; TCGTT TCCTG GGGAT GAGTC; rs765727 Reverse). Fold enrichment was calculated in comparison to the IgG sample: Fold enrichment = 2^-(IgG-Sample). In case of non-detectable signal (>40CT) the fold-enrichment was set to 1. Finally, the 5 donors were genotyped for the SNP rs10958403 (A/G) using the Taqman Genotyping assay, or by amplification and Sanger sequencing of the region around SNP rs765727 (C/T).

siRNA

To assess the role of *SOX17* in hPAECs, siRNA experiments were performed using silencer select siRNA (ThermoFisher) targeting *SOX17* (#s34626). Comparable control siRNA was also used and included two scrambled-siRNA (#ASO2FOQH, #4390847) and a control targeting GAPDH (#ASO2FLIC). hPAECs were seeded in 6 well plates. RNAiMAX (ThermoFisher) was used to transfect hPAECs for 6 hours before replacing the media. 24 hours later, this was removed and 1 ml EGM2 (0.2% FBS) was added to the cells which were incubated overnight. At 48 hours after siRNA exposure, transfected cells were either used for RNA extraction and RT-qPCR, RNAseq analysis, whole-cell protein extraction and western blotting or functional experiments.

RNAseq Analysis

To assess whole transcriptomic effects of knockdown of *SOX17* by siRNA and CRISPR-I *SOX17*-signal 2 and -signal 1, RNA sequencing (RNAseq) was carried out by the Imperial College BRC Genomics Facility. To obtain transcript abundance values, salmon software (version 1.2.1) and GENCODE (release 28) were used to analyse fastq files (50). These were converted to gene expression data using the Bioconductor package tximport (51) (version 1.2.5042, R studio, US) [R Core Team (2018), 2015]. For siRNA samples, gene expression was compared between the negative control condition and the relevant genetic manipulation condition using EdgeR (52). For CRISPR−I samples, gene expression was compared to the BFP control. PC analysis and correction using the FDR method were completed in R Studio and plotted using the EnhancedVolcano package (53) in R studio.

To assess the gene ontology changes resulting from either siRNA-*SOX17*, CRISPR-I *SOX17*-signal 2 or signal 1, over-representation analysis was performed using the WEB-based Gene SeTAnaLysis Toolkit (Webgestalt). For siRNA samples, genes were only included if they met an FDR cut off (q<0.05). Due to the lower effect size of the CRISPR−I analysis, p<0.05 was used instead of q<0.05. The method of interest used was over-representation analysis using the functional database category gene ontology the categories biological processes, molecular function, cellular component and KEGG pathway analysis.

qPCR

To investigate the change in expression of target genes, reverse transcription-PCR (RTPCR) and qPCR was performed with actin beta (ACTβ) used as a reference gene. Primers are shown in Table S5. RTPCR was performed using Multiscribe Reverse Transcriptase (Applied Biosystems, ThermoFisher) and the resulting cDNA was used for qPCR using PowerSYBRgreen mastermix (Applied Biosystems, ThermoFisher). The relative expression of target genes as calculated as

$$2^{-(\delta CT\ [gene]\ \check{}\ \delta CT\ [ACT\beta])}$$

and compared to the relevant experimental control (δCT – delta cycle threshold).

Western Blotting

To assess the levels of Sox17 protein following siRNA transduction, total protein was extracted from cells using RIPA buffer (10X, Sigma) supplemented with protease and phosphatase inhibitor cocktail (ThermoFisher). Proteins were diluted 1:10 and quantified using a Pierce Coomassie (Bradford) assay (ThermoFisher) according to the manufacturer's instructions. Proteins were denatured for 5 minutes at 95°C in Laemmli SDS sample buffer (ThermoFisher) before separating on a NuPAGE 4 to 12%, Nis-TRIS mini protein gel (ThermoFisher) in NuPAGE MES-SDS running buffer (1X, ThermoFisher). Dry transfer was completed using a trans-blot turbo mini PVDF transfer pack and a trans-blot turbo transfer system (both BioRad, USA). Membranes were then blocked in 5% milk (Sigma) in 1x TBST (ThermoFisher) for 1 hour before incubating in primary antibodies (Table S6) overnight at 4°C. The membranes were washed three times for 15 minutes in 1x TBST before incubating for 1 hour at room temperature in the relevant secondary antibody (Table S6). Membranes were then washed three times for 10 minutes in 1x TBST before adding 750µl immobobilon crescendo western HRP substrate (MERCK) and imaging with a ChemiDoc MP imaging system (Universal Hood 3, BioRad) and ImageLab (BioRad). Protein quantification analysis was performed in ImageJ (Version 1.52a, ImageJ, USA).

Proteomic analysis

The methodology of plasma extraction and protein concentration measurement was based on a recent proteomics study by Rhodes et al (16). Patient characteristics are shown in Table S2. Peripheral venous blood was collected during patients' routine clinical appointments. The samples were placed in ice and centrifuged at 1300 x g for 15mins within 30 minutes of collection. Following this, the samples were stored in a freezer at -80°C before protein concentrations were measured. 120 µL of each plasma sample was required for the SomaScan assay which was performed using a SomaScan V4 (SomaLogic Inc, Boulder, CO, USA). The plasma concentrations of 4808 proteins were detected using this assay. Patient clinical data were collected within 30 days of blood sampling and biochemical data were collected within 7 days.

The SNP genotypes for *SOX17* signal 1 (rs13266183) were obtained from the whole genome sequencing study from the UK National Institute for Health Research BioResource (7). Linear regression models were conducted with *SOX17* signal 1 genotypes being the independent variables, protein concentrations as the dependent variables and age and sex included as covariates. The p-values from the linear regression were corrected for multiple comparisons using the false discovery rate (FDR) method. Proteins with a coefficient estimate (β) > 2.5 were classified as upregulated while a β < -2.5 were classed as downregulated. Separate linear regression model was conducted on patients' samples 1 year after baseline as an internal validation. All analyses were completed in R using Rstudio v1.4.1106 and the volcano plots were designed using the package "EnhancedVolcano".

Functional Assays

To assess the effect of siRNA-*SOX17* on hPAEC function, assays investigating proliferation, apoptosis, cell viability, adhesion and barrier function were used and are discussed below.

MTT assay

To assess proliferation of cells, an MTT assay was used. Cells were plated in EGM2 (10% FBS) in 96-well plates at 10,000/well and allowed to grow overnight before commencing with siRNA as described

previously. Following siRNA exposure, VEGF [50 and 100 ng/ml] was added for 24 hours. On the day of the MTT, the EGM2 was replaced, and blank wells were created. 10 µl of MTT [5 mg/ml] reagent was added, and the cells were incubated for 4 hours. Following this, 100µl of MTT detergent (94.65% isopropanol, 5% NP40, 0.35% HCL [1 M]) was added to each well and the plate was shaken for 10 minutes. A plate reader was then used to test absorbance at 570nM.

Caspase 3 assay and CellTitre-Glo assay

To assess apoptosis or viability of cells, the Promega Caspase-Glo 3/7 assay or CellTitre-Glo kit was used. Cells were plated in EGM2 (10% FBS) in 96-well plates at 15,000/well and allowed to grow overnight before commencing with siRNA as described previously. Following siRNA exposure, lipopolysaccharide (LPS) or TNF-α was added for 24 hours. Following this, the caspase assay or CellTitre-Glo kit was performed according to the manufacturer's instructions.

Adhesion assay

To assess the effect of siRNA treatment on proficiency of cell binding onto flat-well cell culture plates, an adhesion assay was performed. Cells are seeded at a density of 10,000 cells/well in 96-well plates coated with nothing or collagen IV (Sigma C6745). After incubation at 37oC for 30 minutes, the wells containing the cells were washed with PBS once, before staining with Coomassie blue for 5 minutes. The stain is then removed from the wells and PBS washes carried out until liquid runs clear. The cells that remained attached to the bottom of the wells were then imaged under a microscope and cell counts were obtained using Image J.

Boyden chamber assay

To assess permeability, a Boyden chamber assay was used. Cells that had undergone siRNA-*SOX17* were plated into Corning 24-well inserts (Corning, US) and left to acclimatise for 24 hours. Following this, IL6 (100ng/ml) was added to the cells for 24 hours. The Boyden chamber was then started with the addition of 40kDa FTIC-dextran (ThermoFisher) to the insert. After 240 minutes, 100ul of EGM2 in duplicate was taken from the bottom chamber and placed into a 96 well plate and the fluorescence was measured with a plate reader. Blank wells, of only EGM2, were also measured.

Electrical cell Substrate Impedance Sensing (ECIS) assays measuring transendothelial resistance.

hPAECs were transfected with NT siRNA or SOX17 siRNA, and 48h after transfection cells were seeded in a 96-well ECIS array, coated with 0.1% gelatin. Directly after seeding, ECIS measurements were initiated (4000Hz, 300sec interval). Medium change was performed 24h after seeding. Separate experiments were performed with three different HPAEC donors.

In-silico analysis using the Connectivity Map

To analyse the differential expression patterns which occur when *SOX17* expression is manipulated, RNA-sequencing of *SOX17*-signal 1 CRISPR-I hPAECs was performed as previously stated. Three test conditions were produced: *SOX17*-promoter repression, *SOX17*-promoter activation and *SOX17*-signal 1 repression. Lists of the genes which were most differentially expressed were created. Only genes that had (log)fold change of over 0.25 (or below -0.25) and $p<0.05$ were used. Gene names were changed to HUGO-approved names using Genenames.org (54) before compiling lists of the top up-regulated and down-regulated genes for each of the three conditions (Table S3). To discover compounds that could be repurposed for the treatment of SOX17 dysfunction, the CMap was used (17). Differential gene expression lists (Table S3) were used to create queries for the three conditions,

SOX17-promoter activation, *SOX17*-promoter repression, and *SOX17*-signal 1 repression. Candidate compounds with a tau score of over 90 or under -90 were selected. For *SOX17*-promoter activation, compounds were chosen with a score of over +90. For *SOX17*-promoter repression and *SOX17* signal-1 repression, compounds were chosen with a score of under -90. These scores were chosen as they represent a connection with an increase in *SOX17* expression or the opposite of a connection with a decrease. The compounds selected were Sirolimus, Aminopurvalanol-a and YK-4279.

300,000 hPAEC per/well of a 6-well plate were plated in EGM2 (10% FBS) and left to acclimatise for 1 day. On day two, media was changed to 0.2% FBS to quiesce the cells. On the day of exposure, media was replaced with EGM2 (10% FBS) before beginning drug exposures. For cell exposure experiments, Aminopurvalanol-A (B6935, APExBIO, Houston, Texas) and YK-4279 (A3946, APExBIO) were used at [10nM] and [50nM]. Sirolimus (A8314, APExBIO) was used at [0.1 nM] and [10 nM]. Cells were exposed to the drugs for both 6 and 24 hours and vehicle (DMSO) controls were also created.

The signature of each compound was compared with the condition signature, for example, the sirolimus signature was compared with *SOX17*-promoter repression. The genes selected for qPCR were consistently perturbed (had a high z-score) in the compound signature across five cell lines or more and were present in the condition gene list. This enabled the selection of highly correlated genes whose gene expression could be assessed to investigate the accuracy of the CMap and allow the assessment of the compound's effect in PAECs (Table S7). BMPR1a and IL1R1a were also selected (when present in both lists) as they are biologically relevant in PAH. mRNA extraction and qRT-PCR were performed as described above.

Generation of the *SOX17* enhancer knockout mice

To determine if loss of the *SOX17* enhancer contributes to the development of PAH, mice lacking the enhancer region containing SOX17 GWAS signal 1 region (Chromosome1: 4,592,560-4,593,307) were generated by CRISPR/Cas9-mediated deletion (SOX17-enhKO) on C57BL/6 background using the Medical Research Council Es Cell and Transgenics facility at Hammersmith Hospital. Mouse epigenomic H3K4m1 data show that this area is also likely to be an active regulatory region in mice. Forward ACAACCAGCTCGGTAAACTT and reverse AAACTTTGTGTCCCTCGTCCT primers were used to test gDNA extracted from ear snips (Quiagen DNA extraction kit) for genotyping: wild type (WT) allele (1829bp) and knockout allele (684bp).

Evans blue-albumin tracer measurement of pulmonary vascular permeability

15 WT (male n=8, female n=7) and 17 SOX17-enhKO (male n=8, female n=9) mice were placed in the hypoxia 10%O2 normobaric chamber for 1-week. Mice were anaesthesized and Evans blue–albumin (EBA) (40 mg/ml BSA with 1% EBA Sigma E2129) was injected into the tail vein at a dose of 8 µl per mouse body weight gram and allowed to circulate in the blood vessels for 60 mins. Intravascular EBA was washed by PBS perfusion from the right ventricle for 2 min. Mouse lungs were then excised, homogenized in 1 ml PBS, and extracted in 2 ml formamide overnight at 60 °C. Evans blue content (optical density) was determined spectrophotometrically at 620 nm of the formamide extract and normalized by lung weight (ug/gm lung ratio) or plasma Evans blue content (lung/plasma %).

Chronic hypoxia induced pulmonary hypertension

WT (male n=8, female n=8) and SOX17-enhKO (male n=14, female n=13) mice were placed in the hypoxia 10% O_2 normobaric chamber, whilst WT (male n=4, female n=3) and SOX17-enhKO (male n=6, female n=6) mice of matching age (9 weeks) were kept in normoxia room air. Right ventricular systolic pressure (RVSP) by direct cardiac puncture and right ventricular hypertrophy (RV/LV+sep ratio,

RV/body weight) were assessed after 3-weeks hypoxia exposure (endpoint). Tissues (Lung and heart) were collected for histological and biochemical examinations. Pulmonary vascular remodeling (muscularization of small intrapulmonary arteries) was determined by counting all muscularized (showing thickened α-SMA-positive media) vessels with a diameter smaller than 50 µm in each lung section and expressed as a percent of total vessel counts (Elastic Van Gieson EVG positive staining). SOX17 protein expression levels in the lung were assessed by western blotting using anti-SOX17 antibody (ab191699, Abcam, 1:1000 dilution) with secondary goat anti-rabbit antibody (A6154, Sigma, 1:2000 dilution). All animal experiments were conducted in accordance with scientific procedures approved by the UK Home Office under Animals Act 1986. The protocols are also approved and reviewed by Imperial College Animal Welfare and Ethical Review Body.

Sugen/Hypoxia (SuHx)-induced PH mouse model

SuHx-PH mouse model consisted of 3 weekly s.c. injections of the Sugen VEGF receptor-2 inhibitor SU5416 (Bio-Techne, Minneapolis, MN) at 20 mg/kg, 10 mg/kg or 5 mg/kg in 100µl DMSO or vehicle alone. During the 3-week SU5416 treatment, mice were exposed to hypoxia (8.5% or 12% O2) or normoxia (Nx) for 3 weeks. Development of PH was determined by measurement of right ventricular systolic pressure (RVSP), right ventricular (RV) hypertrophy (RV to left ventricle + septum wet weight (RV/LV+S) ratio). To measure RVSP, mice were anesthetized via intra-peritoneal injection with ketamine (100 mg/kg) and xylazine (10 mg/kg). RVSP was measured through a trans-thoracic route using a Millar catheter (ADInstruments, Colorado Springs, CO) and data were collected and analyzed using the LabChart Software (ADInstruments).

Endothelial colony forming cells donors

Demographics are provided in ***Table S8.***

Graphing and Statistical Analysis

GraphPad Prism (version 8.0.2, GraphPad Software, San Diego, California, USA) was used for all graphing and statistical analyses except those discussed previously. All graphs show mean (±SEM). R studio (version 1.2.5042, RStudio Inc) was used for all RNAseq and proteomics analysis and graphing of PCA and volcano plots.

Our power calculations for the animal studies indicated that with an expected difference in RVSP of 5 mmHg and a standard deviation of 4 mmHg, a minimum of n=12 in each group would provide 86.5% power to detect a significant difference at alpha=0.05.

Supplemental Figures

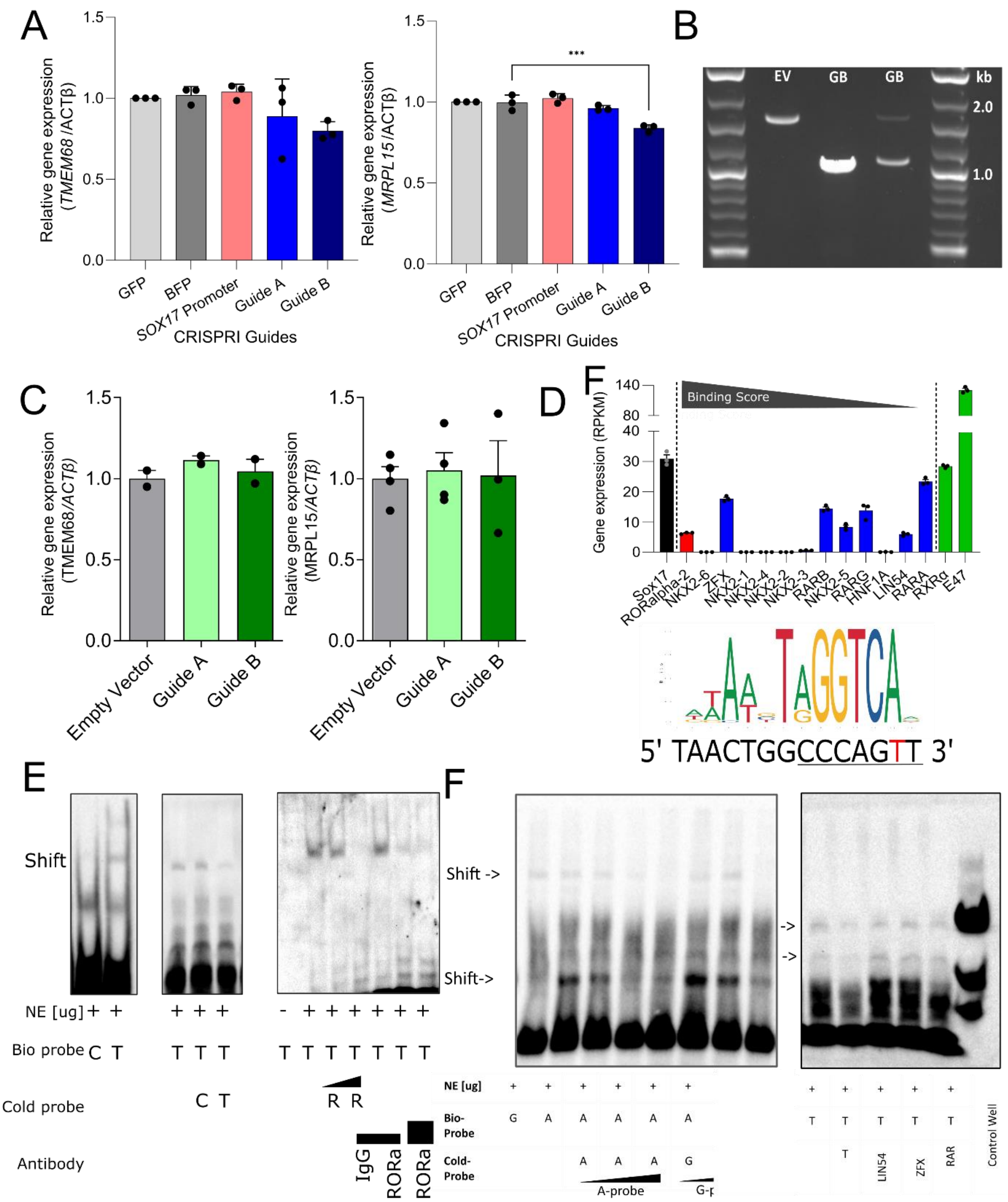

Figure S1) A) Relative gene expression of MRPL15 and TMEM68 compared to ACTβ in hPAEC. Ordinary 1-way ANOVA of conditions compared to BFP condition with Dunnett's multiple comparisons test. * - $p<0.05$. n=3. CRISPR-inhibition guides A/B target *SOX17*-signal 2. GFP, green fluorescent protein. BFP, blue fluorescent protein. **B) Efficiency testing of CRISPR-deletion guides (GBs) versus empty vector (EV).** Gel showing lower weight product of successful cleavage of target site. **C) Effect on gene expression of TMEM68 and MRPL15 following CRISPR-deletion of *SOX17*-signal 1 region.**

EV, empty vector. CRISPR-D guides A/B target *SOX17*-signal 1 for deletion. n=3. **D) The expression (RPKM) of TFs of interest for rs765727 in hPAECs.** *ROR-alpha* is shown in red. *SOX17* is shown in black for reference. Transcription factors found through CIS-BP are shown in blue, those found through PROMO are shown in green. The underlined sequence refers to the potential binding location of *ROR-alpha* and the T in red font is the site of rs765727. **E) EMSA assay showing binding of hPAEC nuclear proteins to 21bp DNA probes containing the sequence at the rs765727 region.** The shift is highlighted. CRISPR-inhibition guides A/B target *SOX17*-signal 2. NE, nuclear extract from hPAECs. Bio-probe, biotin-labelled probe. Cold-Probe, unlabelled probe. C/T, alleles of *SOX17* variants included in probes. R, RORα competitive probe. IgG, Mouse IgG. The black triangles show increasing molecular excess from left to right. RPKM, reads per kilobase of transcript per million mapped reads. The binding score (taken from CIS-BP) refers to the predicted likelihood of the transcription factor binding to the given sequence and decreases from left to right. Representative image of n=3. **F) Transcription factor competitive EMSA for Lin54, ZFX and RAR.** Control well contains control reagents from the manufacturer's kit. NE, nuclear extract from hPAECs. Bio-probe, biotin-labelled probe. Cold-Probe, unlabelled probe. LIN54, ZFX, RAR, competitive probes.

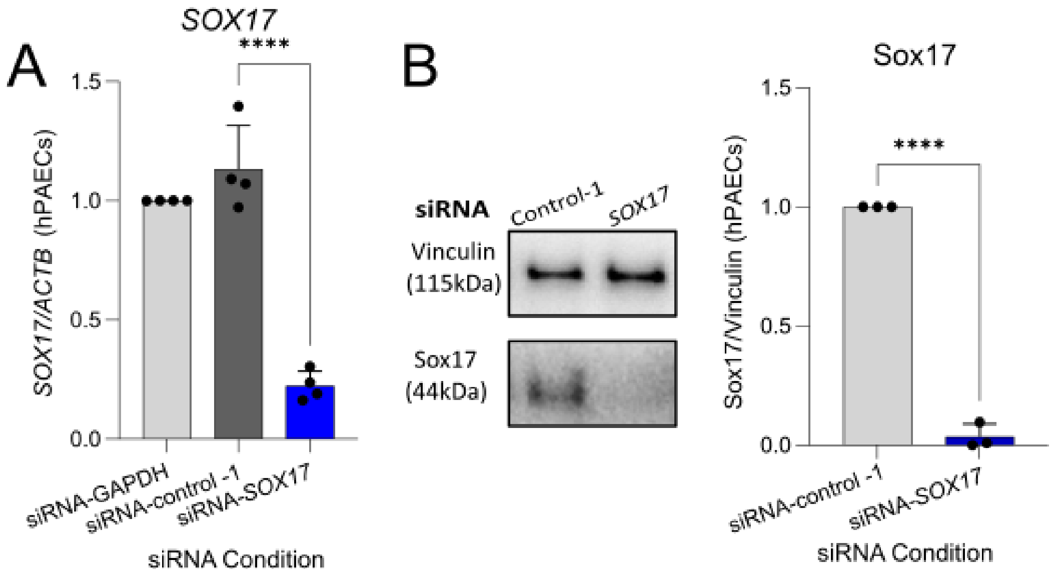

Figure S2) Quantification of Sox17 following siRNA. A) Relative gene expression of *SOX17* by qPCR. The change in *SOX17* expression is normalised to *ACTβ* and all siRNA conditions are relative to the positive control. **B) Protein quantification of Sox17.** Representative image of a western blot of Sox17 and vinculin protein in *SOX17*-siRNA and siRNA-negative samples and quantification of western blotting of Sox17. Sox17 protein level is normalised to vinculin and relative to the negative control. All statistical tests shown in this figure are paired, one-way, student's t test. **** - p<0.0001. n=3 (protein) or n=4 (mRNA).

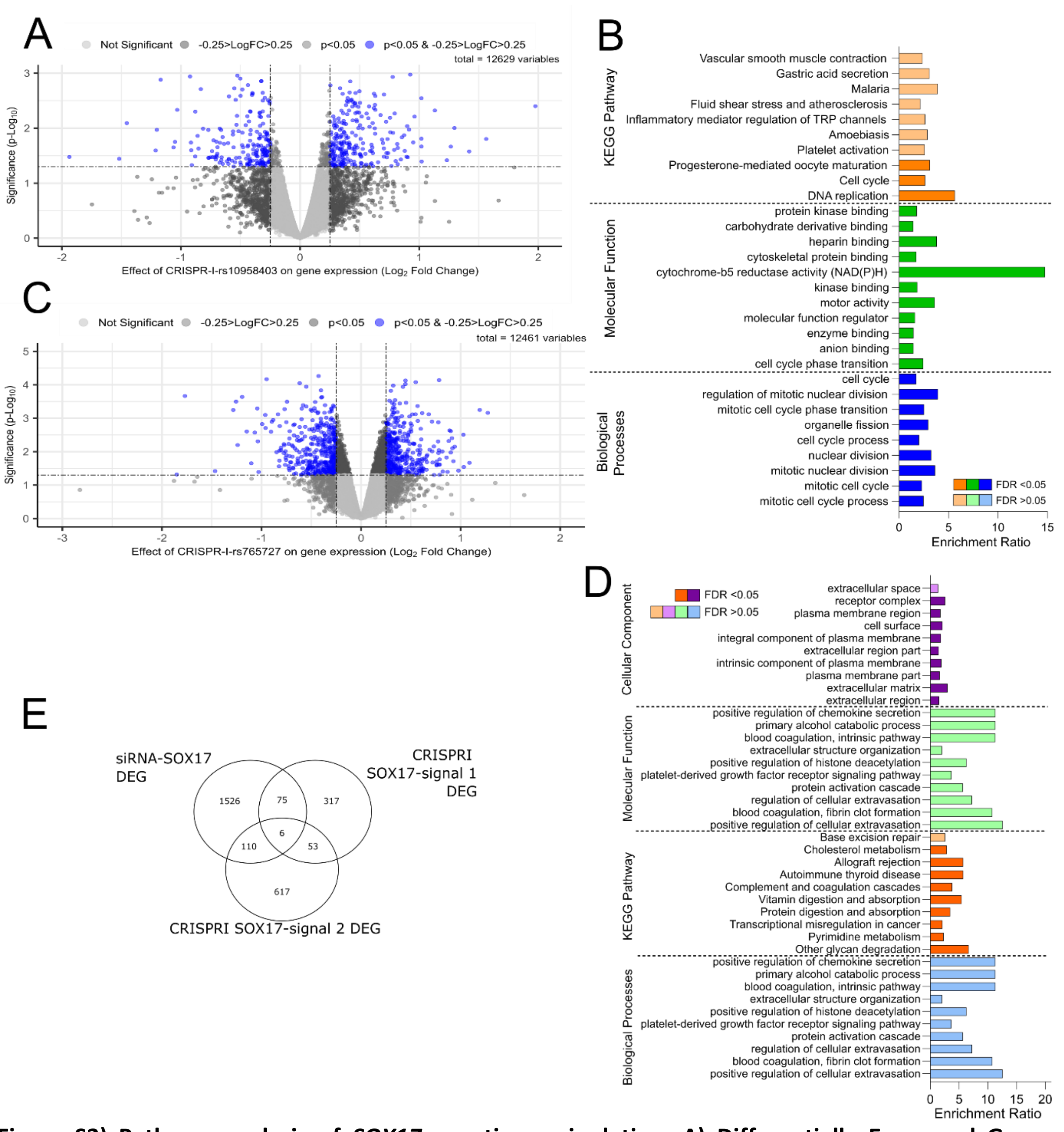

Figure S3) Pathway analysis of *SOX17* genetic manipulation. A) Differentially Expressed Genes following CRISPR-inhibition of *SOX17*-Signal 1 in hPAECs. Volcano plot of the $\log_2$ fold change (FC) between CRISPRI of *SOX17*-Signal 1 and BFP Control and the negative $\log_{10}$ p-value. Differentially expressed genes shown in blue met the cut off points: $p<0.05$ and $\log_2$ fold change <-0.25 or >0.25

n=4, 12629 variables. **B) Over-representation Analysis for enriched pathways and functions following CRISPR-inhibition of *SOX17*-signal 1.** Gene ontology analysis of Cell component (purple), biological process (green), KEGG pathway (orange) and molecular function (blue) enrichment following *SOX17*-signal 1 in hPAECs. Darker colours indicate f<0.05. Lighter colours indicate f>0.05. Enrichment ratios were obtained from WebGestalt. **C) Differentially Expressed Genes following CRISPR-inhibition of *SOX17*-Signal 2 in hPAECs.** Volcano plot of the $\log_2$ fold change in gene expression between hPAEC targeted with CRISPRI of *SOX17*-Signal 2 and a BFP control and the negative $\log_{10}$ p-value. n=4, 12271 variables. **D) Over-representation Analysis for enriched pathways and functions following CRISPR-inhibition of *SOX17*-signal 2.** Gene ontology analysis of Cell component (purple), biological process (green), KEGG pathway (orange) and molecular function (blue) enrichment following *SOX17*-signal 2 in hPAECs. **E) Venn diagram of DEG.** Overlap of DEG from RNAseq analyses of si-SOX17 and CRISPR inhibition of SOX17 signals 1 and 2.

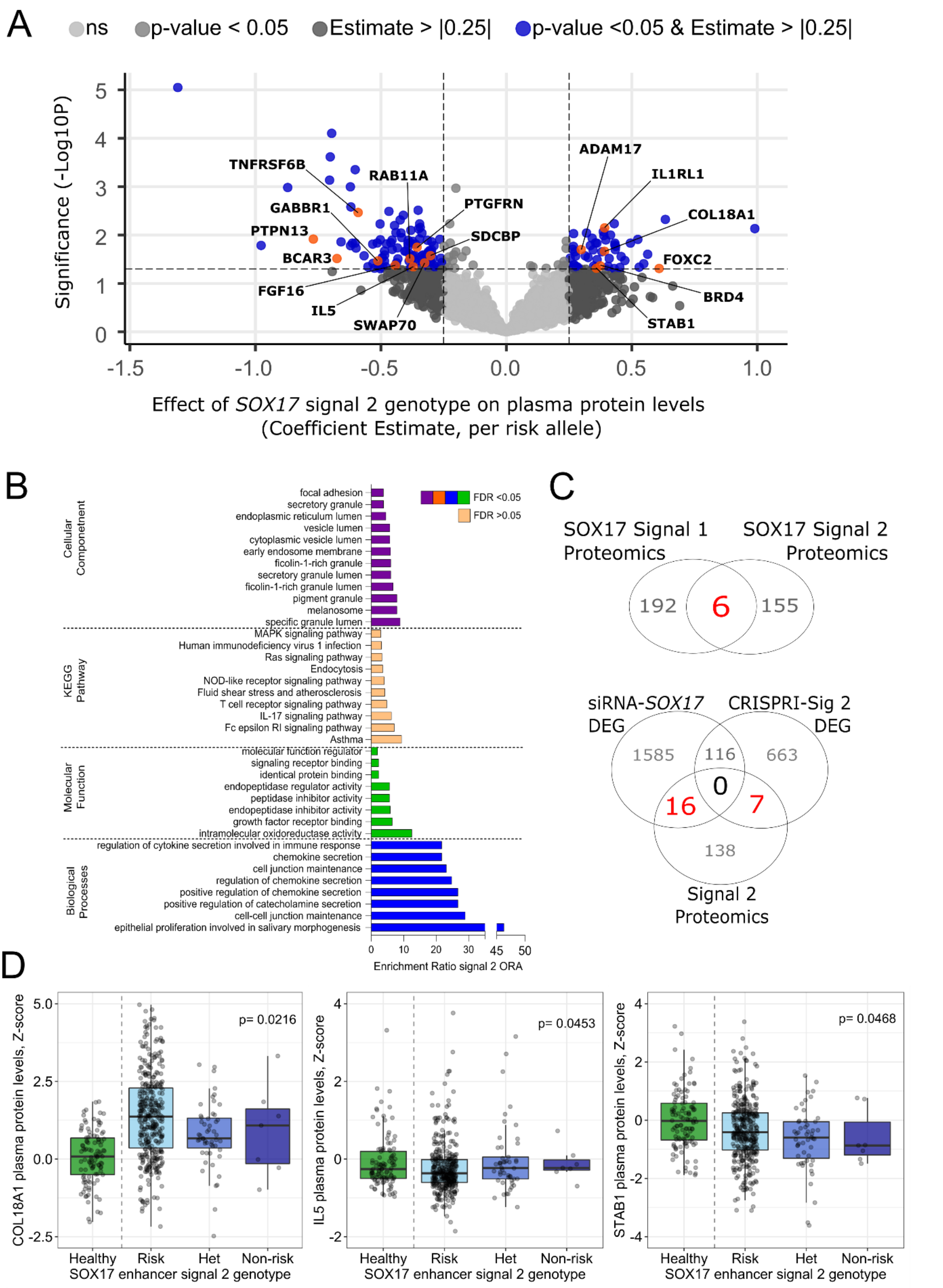

Figure S4) Effect of genotype on patient proteomics. A) Linear regression for the effect of *SOX17*-signal 2 on the levels of serum proteins in patient sample. Volcano plot of the coefficient estimate and the negative $\log_{10}$ p-value. Corrected for age and sex. Protein shown in blue met threshold b-estimate > |0.25| and p < 0.05. Protiens of interest are labelled and shown in orange. n =4349**. B) GO analysis for the significantly affected by signal 2 genotype proteins**. Gene ontology analysis of Cell

component (purple), biological process (green), KEGG pathway (orange) and molecular function (blue) enrichment. Proteins met the threshold b-estimate > |0.25| and $p < 0.05$. Enrichment ratios were obtained from WebGestalt. **C) Comparisons of transcriptomic and proteomic analysis.** Venn diagram showing overlapping differentially expressed genes and proteins from transcriptomic and proteomic analysis from *SOX17*-signal 1 and *SOX17*-signal 2 proteomis analysis (upper) and CRISPRi of *SOX17*-signal 2, siRNA-*SOX17* and *SOX17*- signal 2 enhancer variant genotype on patient proteomics (lower). DEG, differentially expressed genes. Numbers in red show genes and/or proteins which are in common between all analyses. **D) Z-scored proteins in healthy controls versus patients with different genotypes.** Proteins are COL18A1, IL5 and STAB1. Risk, homozygous for PAH-associated allele. Non-risk, homozygous for non-risk allele. Het, heterozygotes. Control, n=108. Risk enhancer, n=373. Het signal 2, n=51. Non-risk, n=7.

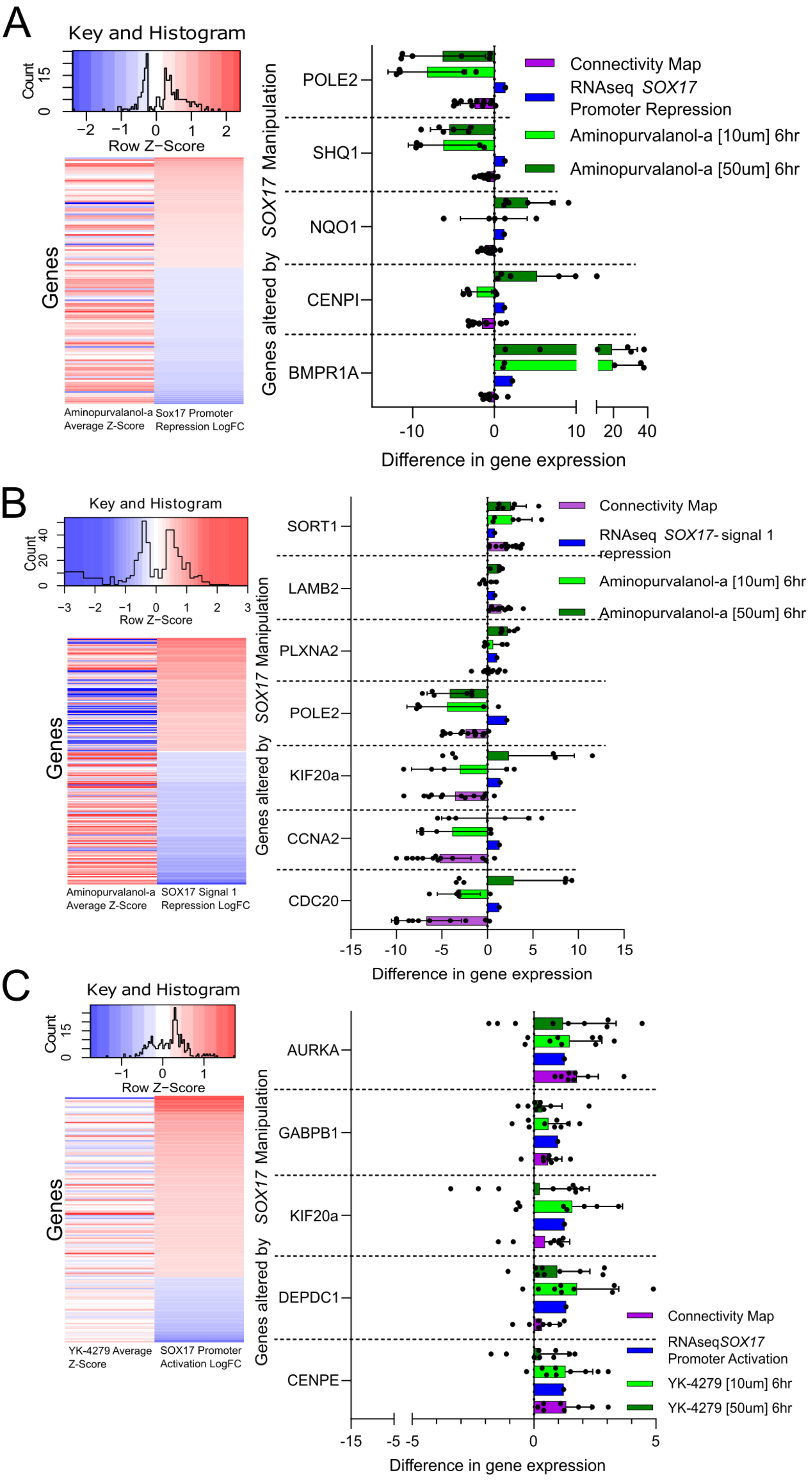
A
Key and Histogram
Count
Row Z−Score
Genes
Aminopurvalanol-a Average Z-Score
Sox17 Promoter Repression LogFC
Genes altered by SOX17 Manipulation
POLE2
SHQ1
NQO1
CENPI
BMPR1A
Difference in gene expression
Connectivity Map
RNAseq SOX17 Promoter Repression
Aminopurvalanol-a [10um] 6hr
Aminopurvalanol-a [50um] 6hr
B
Key and Histogram
Count
Row Z−Score
Genes
Aminopurvalanol-a Average Z-Score
SOX17 Signal 1 Repression LogFC
Genes altered by SOX17 Manipulation
SORT1
LAMB2
PLXNA2
POLE2
KIF20a
CCNA2
CDC20
Difference in gene expression
Connectivity Map
RNAseq SOX17- signal 1 repression
Aminopurvalanol-a [10um] 6hr
Aminopurvalanol-a [50um] 6hr
C
Key and Histogram
Count
Row Z−Score
Genes
YK-4279 Average Z-Score
SOX17 Promoter Activation LogFC
Genes altered by SOX17 Manipulation
AURKA
GABPB1
KIF20a
DEPDC1
CENPE
Difference in gene expression
Connectivity Map
RNAseqSOX17 Promoter Activation
YK-4279 [10um] 6hr
YK-4279 [50um] 6hr

Figure S5) Repurposing of Compounds to Rescue loss of SOX17 Function in PAH. A) Heatmap of *SOX17* promoter CRISPR-I Log_{10} fold change and Aminopurvalanol-a perturbagen Z-scores of genes used in CMap Query. Differentially Expressed Genes Following Aminopurvalanol-a Exposure in hPAECs. Gene expression of *POLE2, SHQ1, NQO1, CENPI* and *BMPR1a*. Connectivity map, perturbagen z-score taken from the CMap. RNAseq *SOX17* promoter repression via CRISPRI (n=3), fold change from RNAseq analysis of DEG following CRISPRI of the *SOX17* promoter (n=3). Aminopurvalanol-a [10µm/50 µm] (n=3), the change in target gene expression is normalised to *ACTβ* and all siRNA conditions are relative to the GAPDH-targeting siRNA control in hPAECs following Aminopurvalanol exposure at the stated concentrations. **B) Heatmap of *SOX17*-signal 1 CRISPR-I Log_{10} fold change and Aminopurvalanol-a perturbagen Z-scores of genes used in CMap Query.** Differentially Expressed Genes Following Aminopurvalanol-a Exposure in hPAECs. Gene expression of *SORT1, LAMB2, PLXNA2, POLE2, KIF20a, CCNA2* and *CDC20*. Connectivity map, perturbagen z-score taken from the CMap. RNAseq *SOX17*-signal 2 repression via CRISPRI (n=3), fold change from RNAseq analysis of DEG following CRISPRI of the *SOX17*-signal 2 (n=3). Aminopurvalanol-a [10µm/50 µm] (n=3),, the change in target gene expression is normalised to *ACTβ* and all siRNA conditions are relative to the GAPDH-targeting siRNA control in hPAECs following Aminopurvalanol exposure at the stated concentrations. **C) Heatmap of *SOX17* promoter CRISPR-a Log_{10} fold change and YK-4279 perturbagen Z-scores of genes used in CMap Query.** Differentially Expressed Genes Following YK-4279 Exposure in hPAECs. Gene expression of *SORT1, LAMB2, PLXNA2, POLE2, KIF20a, CCNA2* and *CDC20*. Connectivity map, perturbagen z-score taken from the CMap. RNAseq *SOX17* promoter activation via CRISPRA (n=3), fold change from RNAseq analysis of DEG following CRISPRa of the *SOX17* promoter (n=3). YK-4279 [10µm/50 µm] (n=3), the change in target gene expression is normalised to *ACTβ* and all siRNA conditions are relative to the GAPDH-targeting siRNA control in hPAECs following YK-4279 exposure at the stated concentrations.

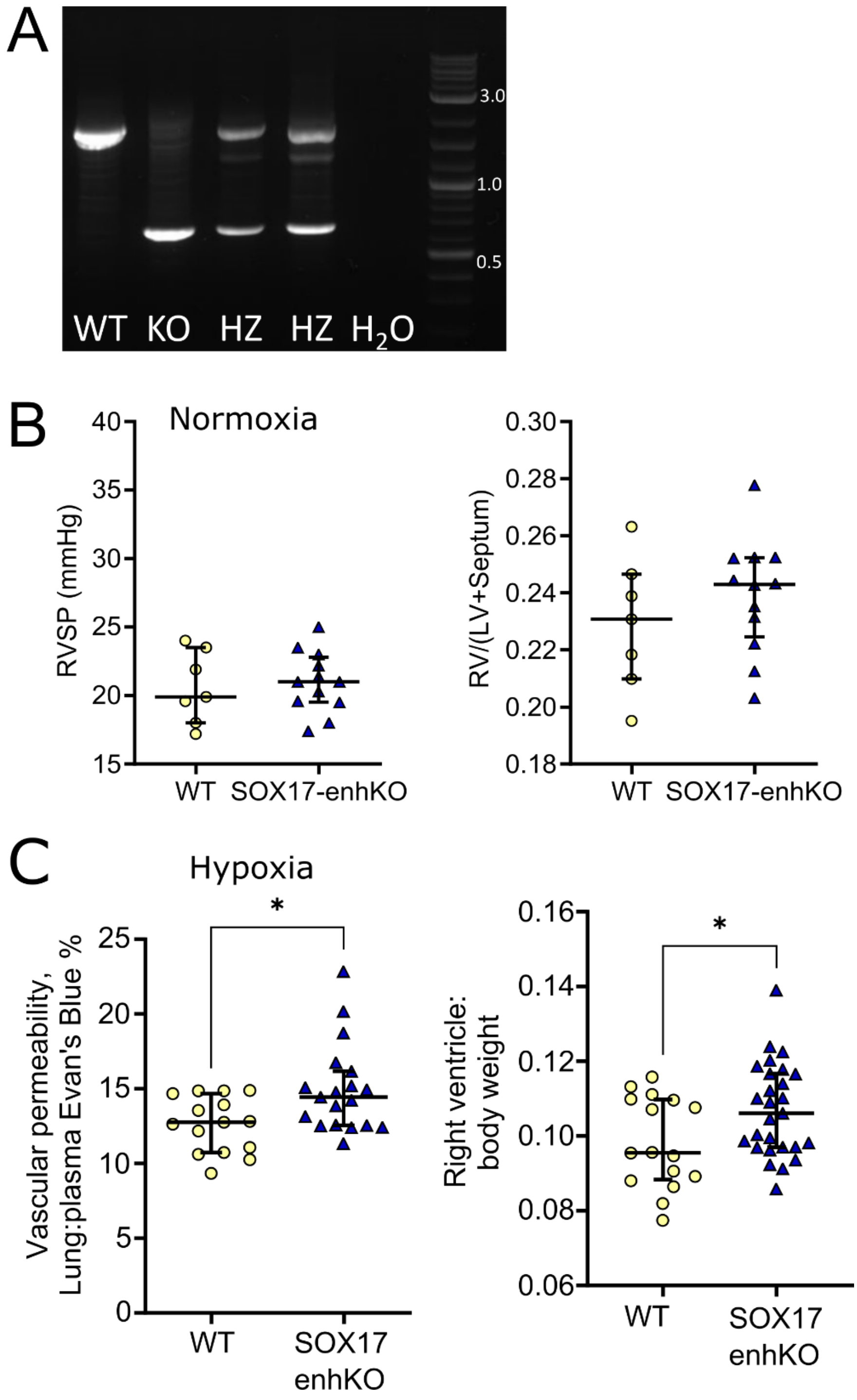

Figure S6) Genotyping and normoxic phenotyping of *SOX17* enhancer knockout mice model. A) Genotyping of *SOX17* enhancer knockout mice. The right-hand well shows a ladder with labels for 3, 1 and 0.5 kb. WT, wild type. KO, knockout. HZ, heterozygotes. **B) Normoxia Haemodynamic measurements of *SOX17* enhancer knockout mice.** Right ventricular systolic pressure (RVSP) and right ventricular hypertrophy index (RVH, RV/LV+sep). n=7 and n=12 . **C) Hypoxia vascular permeability and RVH measurements of SOX17 enhancer knockout mice following 3 weeks hypoxia 10% O_2**. *-p<0.05, versus WT (unpaired t-tests). n=15/16 and n=19/27.

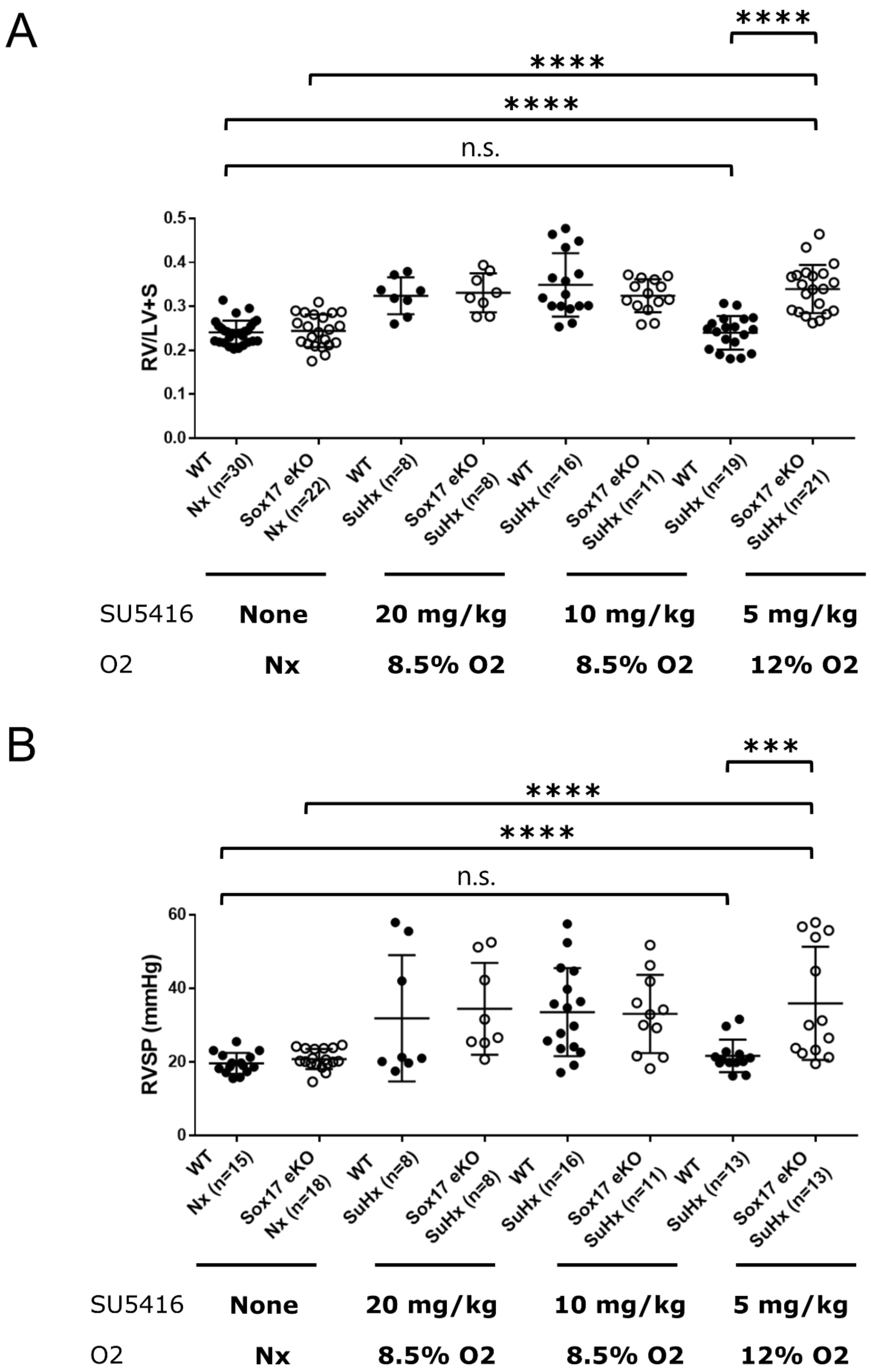

Figure S7) Phenotyping of *SOX17* enhancer knockout mice model (eKO) to different levels of Sugen-5416 and hypoxia. A) Right ventricular hypertrophy index (RV / LV + S). B) Right ventricular systolic pressure (RVSP mmHg). SuHx, Sugen-5416 Hypoxia. O2, oxygen (Nx=normoxia). Numbers are shown on graphs. Ordinary One-way ANOVA. WT, wild type. KO, knockout.

Supplemental Tables

Table S1: CRISPR Guide Sequences

Target	FWD Guide ('5-3')	REV Guide ('5-3')
SOX17 promoter	-CACCGTCTGGTCTACAGCGTACCC-	-AAACGGGTACGCTGTAGACCAGAC-
SOX17-Signal 2 guide A	- CACCGCTGGCCCAGCTCAAGTTACT -	- AAACAGTAACTTGAGCTGGGCCAGC -
SOX17-Signal 2 guide B	- CACCGGAGGCCAAGTAACTTGAGC -	- AAACGCTCAAGTTACTTGGCCTCC -
SOX17-Signal 1 Del Guide A	-CGACGTCTCTcaccgGCTTTTCAAAA TTCTTGAAGGTTTTAGAGCTAGAAA TAGCAA-	-GGTCGTCTCCaaacTCCGTGGATA GACTGCGCCAGGGAAAGAGTGGTC TCA-
SOX17-Signal 1 Del Guide B	-CGACGTCTCTcaccgTGCAGC GTCGCTTCTATTTTGTTTTAGA GCTAGAAATAGCAA-	-GGTCGTCTCCaaacCCCGTGGATAG ACTGCGCCAGGGAAAGAGTGGTCTCA-

Table S2) Characteristics of the plasma proteomics study population at sample collection.

**Patient Baseline Characteristics**		
Age at diagnosis, years		44.9 (33.8 - 80.2)
Age at participation, years		51.5 (40.5 – 64.0)
Sex	Male	121 (28.0%)
	Female	310 (72.0%)
Ethnic Origin	White	380 (88.2%)
	Asian	28 (6.5%)
	Black	7 (1.6%)
	Other ethnicity or not stated	16 (3.7%)
PAH Aetiology	IPAH	386 (90.0%)
	HPAH	38 (8.8%)
	Other	7 (1.6%)
NYHA FC	Class I	7 (1.6%)
	Class II	77 (17.9%)
	Class III	277 (64.3%)
	Class IV	56 (13.0%)
	Unknown	14 (3.2%)
6 minute walk distance, m		339 (235 – 416)
mPAP, mmHg		54 (46.0 - 61.3)
mRAP, mmHg		9 (6 - 12)
PAWP, mmHg		10 (7 - 12)
PVR, dynes • sec/cm^5		927.9 (625.4 - 1289.3)*
Cardiac Output, L/min		3.7 (3.0 – 4.8)
Treatment Naïve		138 (32.0%)
Monotherapy	Prostanoid Analogue	11 (2.6%)
	PDE5 inhibitors	61 (14.2%)
	ERA	51 (11.9%)
	Riociguat	1 (0.2%)
Polytherapy		169 (39.2%)$^{\pm}$

Centre of Sample Collection	Glasgow	47
	Imperial and Hammersmith	149
	Newcastle Freeman	38
	Papworth	75
	Royal Brompton	30
	Royal Free	21
	Sheffield	71

Data are in median (IQR), n (%) or n. NYHA FC = New York Heart Association Functional Class. mRAP = mean right atrial pressure. PAWP = pulmonary artery wedge pressure. PDE5 = Phosphodiesterase 5 inhibitors. ERA = Endothelin Receptor Antagonists. N=431

* PVR data were only available for 368 patients. [±] Polytherapy drugs were the same as monotherapy

Table S3) Gene lists used for connectivity map analysis.

Condition	*Upregulated Gene Names*	***Downregulated Gene Names***
SOX17-signal 1 repression	EIF3CL, TRG-AS1, MEG8, MINDY4, RAB3C, GALNT12, POLE2, APCDD1L, ELFN2, ESPN, FANCD2, BARD1, KIF15, FER1L4, IFITM1, CHCHD5, ZNF471, KIF19, LAMA2, FERMT1, FGL2, ATP2A3, LRRC4, ATG9B, ZEB2, ZNF10, ANO2, TMEM156, ZFHX4-AS1, FANCC, APIP, RCAN3, SPC24, ZNF653, SCN3B, DNA2, APOLD1, STC1, DGCR11, PPM1L, SKA3, MAPK13, NME1-NME2, MCEE, HELLS, CYB5RL, TRBC2, PLVAP, CBS, CENPI, CDKN3, THAP10, PRIM1, ZNF559, AHI1, PCBD2, SULT1A1, ORC1, ACKR1, MMS22L, BBS5, GZF1, SPATA13, PBK, FAM111B, THAP6, ZNF283, ZFYVE16, CPXM1, MTRF1, RAD51, CDCA5, C15orf41, DHH, CACFD1, PTCD1, DEPDC1B, DNAJC25-GNG10, DEPDC5, CENPF, KIF2C, CLBA1, KIF20A, CD68, PARPBP, CCNB2, PIMREG, RRM2, PLEKHM3, ARHGAP11A, CXCL6, SLC25A16, BICRA, LMNB1, LINS1, RNASEH2A, TOX, ZWINT, BIRC5, ORC6, C1orf112, SHC3, TMEM97, SPIN4, TNFRSF6B, DLGAP5, RGS5, MRC1, DEPDC1, CENPW, IGFBP3, MGARP, CXCL1, MYBL2, TNRC6B, CEP55, CDCA2, SLC17A9, TTK, MAD2L1, SIPA1L1, PCLAF, KNTC1, SULT1A4, CCNA2, MTO1, PRC1, MCUR1, TRIP13, PTCD2, KLHL8, SCLT1, ITGAE, COL13A1, TK1, SPAAR, UBE2E2, SDC1, PHF19, PTPN2, CDC20, PTTG1, NDC80, IL1RL1, AP5S1, PLCL1, FGFR1OP, DENND4A, FAM76B, FEN1.	IKBKGP1, CDRT4, TMEM132B, TLX1, UPK3BL1, DOCK8, NEBL, ZC3H6, C2CD4A, MAN1B1-DT, HYPK, TRPS1, KCNC3, POU2F1, LGALSL, CAPS, TECTA, FGFR4, TBXA2R, ABCB1, FAM156B, PFKFB2, SLC25A35, C1orf50, TRIM45, MITF, PLA2G6, DNAH14, POU6F1, HSD11B1L, INHBA, BTBD11, SGIP1, NEUROD2, SHROOM1, IGF2, GLT8D2, SATB2, DACH1, AXIN2, HLX, GOLGA8B, HSPA2, ZNF862, TRIM62, PKD1P1, WDR91, ARG2, CHRFAM7A, ALDH1A1, IQGAP2, FASN, PKD1, PLCG2, ITPR1, TRO, POR, NIPAL2, CD34, PLA2G4C, IKZF5, SELE, CYB5R4, CCL2, SLC25A42, NPR2, CASKIN2, ZNF446, SLC9A7, ZBTB7B, SPTBN5, PGGT1B, MKS1, ACSM3, FRS2, GAB1, SLC26A6, ICOSLG, UNKL, ZNF780A, STC2, PLXNA3, RNF217, CTXN1, SBF2, PHF8, ZHX2, ADPRH, TRAF5, ADARB1, RBMS2, PHC3, KLHL17, CRTC1, PGF, CRACR2B, PIBF1, PIAS4, BCL9, CHST1, SETD1A, ZFX, ARL5A, ICAM1, THBS1, DEPP1, MAPK8, HERC2P9, PITPNM1, ARGLU1, BMP4, DLL4, MT-ND5, PER1, GNAI1, SLC2A6, CPNE5, SORT1, STIM1, UBE2Q1, TRIM69, MT-CYB, EIF4ENIF1, HERC2P3, SART3, USP20, SLC25A23, IPO13, PGGHG, JUP, MGP, PIGQ, RGS19, C12orf65, MAD1L1, PIK3CD, VASH1, AGO4, FABP5, RELB, MYCBP2, RHOB, LAMB2, LAMC1, MCOLN1, SMAD1, INSR, APPBP2, MRTFA, PLXNB1.
SOX17 promoter activation	INO80B-WBP1, RIMS1, RPS4Y1, GJA5, STAC, SEMA3G, KDM5D, USP9Y, IL1A, CD69, SOX17, TGFBI, NPTX2, SLC16A9, DOCK7, CXCL3, PPP1R12B, PTPN7, CXCL1, GJA4, CXCL2, DNA2, TCF15, ZNF519, DIO2, MYRF, LRP4, UNC5B, SELE, HEY1, TCTEX1D1, CCNA1, FST, POLR2J4, VCAM1, TMEM79, ADAM12, NOSTRIN, DCLK1, SDR42E1, KBTBD3, ARHGAP28, CDC6, JAM2, LIPG, APOL4, INHBA, JMJD7, CCDC15, PHGDH, PPARGC1B, INSR, LFNG, PLLP, ZNF585A, CCL2, RFTN2, DTL, TUSC3, CKAP2L, HSPA2, PLK4, ELFN2, ALG14, LYPD1, ACKR3, RND1, DEPDC1, MMS22L, ARHGEF25, ZBTB14, EFNB2, PODN, TTK, PAPLN, DTNA, NRN1, PEG10, GPRIN3, PSRC1, TTC4, CHEK2, IL17RA, COL3A1, ZNF229, TOX, XRRA1, BUB1B, NUF2, NR5A2, GPHN, ZNF383, ACSM3, MYLK, RUNX1, TTC26, KIF20A, CXCL6, COL4A1, IL1RAP, MYBL2, BLM, AURKA, TNFAIP2, ITM2A, MCM5, E2F1, MATN3, TAPT1, SPAG5, RAD51AP1, BIRC3, F2RL1, SPC25, HJURP, NFKBIZ, KSR2, CHST1, TSPAN13, TULP4, MIS18A, ADGRG6, E2F8, CCNB2, RIPK2, ZWINT,	CDC14B, KCNMA1, MSMP, KCND1, SAMD12, IL17RE, ACP5, RAG1, RGPD2, H4C9, FAM74A4, FAM27E3, GRIK5, ZNF490, ZNF571, LRRC34, WASH7P, NOTCH3, FAM221A, ACTR3B, GTF2IRD2, ZNF653, ITGB4, PCDHA10, GALNT8, NEK10, CCL14, RARRES1, ZNF607, NTM, VGLL3, FOXO6, PHLPP1, SESN3, H2AC6, PTGIR, TM7SF2, C1orf56, PDK4, PDE2A, GALNT15, GALNTL5, SLX1B-SULT1A4, SEMA4F, SLC4A8, SLC40A1, MREG, ZNF767P, CHD2, RCCD1, ADAMTS18, RNASET2, STAG3L5P, MMAA, USP49, PHC3, OCLN, DEPDC5, HYAL3, GABPB2, GABPB1, GSTM2, FAM78A, HID1, NPR1, MYEOV, MTURN, FAM217B, TIAM2, HEATR6, EPHA4, ARID4A, WDR90, SCN1B, CDKN2B, IL17D, ZNF286A, SNED1, THBD, ITGA10.

	GORAB, UBE2S, SORBS2, KIF26A, ZWILCH, SKA3, SLC7A11, CENPE, SERPINE2, AURKB, IL3RA, ITGB8, ANLN, NOTCH4, NOCT, GINS1, ABL2, ADAMTS6, DLGAP5, ADAM23, SOX13, IKBKE, GSTCD, RNASE1.	
SOX17 promoter repression	BMPR1A, MEGF10, NPIPA7, LINC01002, RAB3C, CPA4, PPT2-EGFL8, MSMP, ZIC1, ZBTB43, ADCYAP1R1, SH2D2A, THAP10, CD36, GABRE, DRAXIN, SERHL2, LLGL2, ACP5, PSG4, ZNF774, TRBC2, GNG2, APCDD1L, CDO1, PTCH1, RELN, KRT15, ZNF26, FSD1L, LTB4R, TEDC2, CDT1, BAALC, CPNE7, CCBE1, IMMP2L, IL1RL1, PIK3CG, ZNF283, MAPK13, POLE2, MFSD2A, LYVE1, BDH1, DENND1B, ZNF324B, STC1, SHQ1, PTPN22, KRT19, COQ8B, MAP3K6, DMXL2, AKAP9, ADIRF, CCDC68, TNFRSF6B, TREX1, LETM2, NAA16, ADAMTS18, BBS5, TMEM273, ZNF616, CENPJ, MGARP, SMYD4, CRCP, STRIP2, VPS13B, EGFR, ULK3, SLC4A8, TBC1D2, PDE2A, CENPI, EPC1, COL4A6, DDHD1, ADAMTS1, ZNF91, NEGR1, GFPT2, CSPP1, HELLS, MFSD14C, RAB8A, DAGLB, GAS8, ITGAE, NQO1, NBPF14, ATG16L2, NCR3LG1, ZNF280D, FAH, DIS3L.	BACH2, SEMA3G, SOX17, RGPD1, AFAP1L2, SCAPER, LARGE1, MATN2, TBC1D32, TMEM117, C2CD4A, RBPMS2, CX3CL1, CCL2, ZNF850, MGAT5B, SLC15A3, RASA4B, POU6F1, C16orf95, HDAC9, CTSS, OPLAH, HSPA2, PKHD1L1, GJA5, SEMA4D, RAI2, COL3A1, BMF, GDPGP1, SHISA3, NIBAN1, IL3RA, BTBD11, CTSK, APBA1, MXD3, SEMA7A, PITPNM1, SHROOM1, EPHB1, LYPD1, ATXN7L1, CPA3, PAPLN, HOXA-AS2, CHN2, GTF2IRD2, CD34, ATP7B, TBL1XR1, ITGA1, FBXO32, PREX2, MCTP1, FILIP1, NAT1, SLC6A2, PKD1P1, A2M, CEP162, HERC2P3, TCIM, ERCC4, VWA1, CXCL2, PLLP, SEMA4F, EHD3, NTN4, MAMLD1, COMMD3-BMI1, ZFX, ALS2CL, TNFSF10, ACKR3, LAMP3, POMT2, ZNF75A, ATPAF2, EDN1, FANCG, ALDH1A1, AIF1L, ABLIM1, RGS3, COLEC12, C15orf54, KLRG1, WARS1, ZSCAN18, GBP4, SLC2A8, COL8A1, FABP4, TMC6, NYNRIN, PDGFB, LZTS3, SPNS2, BMP4, PRR5L, FAM117A, MMP2, LIG1.

Table S4: EMSA Biotin (bio) Labelled, Unlabeled (cold) and Transcription Factor Competition Probe Sequences.

Signal	Allele	FWD Probe (5’-3’)	REV Probe (5’-3’)
**Biotin-labelled Probes**			
1	G	-TGTCACGTTGCCATAGCATAG GTGCTATTTAGAGCCAGGAA-bio-	-TTCCTGGCTCTAAATAGCACCTAT -GCTATGGCAACGTGACA-bio-
1	A	-TGTCACGTTGCCATAGCATAA GTGCTATTTAGAGCCAGGAA-bio-	TTCCTGGCTCTAAATAGCACTTAT GCTATGGCAACGTGACA-bio-
2	C	-ACTGGCCCAGCTCAAGTTACT-bio-	-AGTAACTTGAGCTGGGCCAGT-bio-
2	T	-ACTGGCCCAGTTCAAGTTACT-bio-	-AGTAACTTGAACTGGGCCAGT-bio-
**Unlabeled (cold) Probes**			
1	G	-TGTCACGTTGCCATAGCATAG GTGCTATTTAGAGCCAGGAA-	-TTCCTGGCTCTAAATAGCACCTAT GCTATGGCAACGTGACA-
1	A	-TGTCACGTTGCCATAGCATAA GTGCTATTTAGAGCCAGGAA-	-TTCCTGGCTCTAAATAGCACTTAT GCTATGGCAACGTGACA-
2	C	-ACTGGCCCAGCTCAAGTTACT-	-AGTAACTTGAGCTGGGCCAGT-
2	T	-ACTGGCCCAGTTCAAGTTACT-	-AGTAACTTGAACTGGGCCAGT-
**Transcription Factor Competition Probes**			
1	HOX-A5	-TGTCACGTTGCCATACACTAAT TGCTATTTAGAGCCAGGAA-	-TTCCTGGCTCTAAATAGCAAT TAGTGTATGGCAACGTGACA-
2	ZFX	-ACTGGCTCAAGTTAAGTTACT-	-AGTAACTTAACTTGAGCCAGT-
2	LIN54	-ACTGGCGCCCAGTTAGTTACT-	-AGTAACTAACTGGGCGCCAGT-
2	ROR-α2	-ACTGGCCAGGTCAAAGTTACT-	-AGTAACTTTGACCTGGCCAGT-
2	RAR	-ACTGGCAGTTCAAGAGTTACT-	-AGTAACTCTTGAACTGCCAGT-

Table S5: qPCR Primer Sequences

Gene	FWD Primer ('5-3')	REV Primer ('5-3')
SOX17	- GGACCGCACGGAATTTGAAC -	- GGACACCACCGAGGAAATGG -
MRPL15	- GCGGATCCTGCCAAATTTCC -	- AACTCTGCTTCCTTGGACGG -
TMEM68	- GCCGTTTGGCATGGTTATGA -	- GGGTCGCCTAAATAGGTCCG -
ADAMTS12	-TTGCTTTGAAGGCGGCAAC-	-CCCAATGTCAACATAACCAGATCC-
MMP17	-CCTCAGCCTGGGAGTGGAGTG-	-GAGCAGCGTGGGGTTTTCAT-
LAMB3	-CAAACATGCGGTGGTGATGT-	-GTGTAGTCAGACGAGCACTCA-
VECAD	-CTTCACCCAGACCAAGTACACA-	-AATGGTGAAAGCGTCCTGGT-
ACTB1	- GCACCACACCTTCTACAATGA -	- GTCATCTTCTCGCGGTTGGC -

Table S6: Antibodies

Protein	Experiment	Antibody Number	Company	Concentration
Sox17	Western Blotting	ab224637	Abcam	1:500
ROR-α	Western Blotting	SC-518081	Santa Cruz	1:100
B-Actin	Western Blotting	SC-47778	Santa Cruz	1:250
Vinculin	Western Blotting	V9131	Sigma	1:250
Anti-Mouse	Western Blotting	ab6728	Abcam	1:2000
Anti-Rabbit	Western Blotting	A6154	Sigma	1:5000
Anti-Goat	Western Blotting	ab6741	Abcam	1:2000
HOXa5	EMSA	sc-515309	Santa Cruz	1ug
ROR-α	EMSA	SC-518081X	Santa Cruz	1ug

Table S7) Gene, Drug, Condition and Primer information for Connectivity Map target genes.

Gene Name	Drug	Condition	FWD Primer (5'-3')	REV Primer (5'-3')
ITGAE	Sirolimus	*SOX17*-promoter repression	-GAGGTCATCTGCTCATGTTTCAGT-	-TTGAAGGGTGTGGGTCTCGT-
RAB8A	Sirolimus	*SOX17*-promoter repression	-ACGGTTTCGGACGATCACAA-	-TCCAGTTCCGGATGTTGTCG-
FAH	Sirolimus	*SOX17*-promoter repression	-TTCACGGTGAGACCAAAAGTCAG-	-GCTTGATGATGCTGAGGTCCA-
PKD1P1	Sirolimus	*SOX17*-promoter repression	-CGACAGCTGCCTGGACTCAT-	-TAGAGCCCTCACCTCAGCGT-
MXD3	Sirolimus	*SOX17*-promoter repression	-GAGCATGGTTATGCGTCCCT-	-CCTGCGCTTCTCCAGTTCAT-
PITPNM1	Sirolimus	*SOX17*-promoter repression	-CTTTGATGCCCACGAAGGCT-	-TTTAGCTGCCTCGGCTCCAG-
MAMLD1	Sirolimus	*SOX17*-promoter repression	-CAGGGGGTGATCTGCAACAG-	-TGACTCTCCAAGGCGTTTCC-
IL1RL1	Sirolimus	*SOX17*-promoter repression	-AGAGGAAAACAAACCCACAAGG-	-CTGGCCGGTGACATTACAGAT-
BMPR1A	Sirolimus	*SOX17*-promoter repression	-GAAAAAGTGGCGGTGAAAGT-	-TAGAGCTGAGTCCAGGAACC-
	Aminopurvalanol-a	*SOX17*-promoter repression		
CENPI	Aminopurvalanol-a	*SOX17*-promoter repression	-GCCTCATCTCCAGGCTTTGT-	-CCGGTTTCTTTGCTTCACGG-
NQO1	Aminopurvalanol-a	*SOX17*-promoter repression	-AGGCTGGTTTGAGCGAGTGT-	-ATGTCCCCGTGGATCCCTTG-
SHQ1	Aminopurvalanol-a	*SOX17*-promoter repression	-TCAGGAAAATGACCCAGCGT-	-AGCTGGGCCTTTGTAAGGGA-
POLE2	Aminopurvalanol-a	*SOX17*-promoter repression	-TCACCAGCACCTCCAACCTG-	-CCTCTGGACCAGGTACAAACACA-
	Aminopurvalanol-a	Signal-1 repression		
ACKR3	Aminopurvalanol-a	*SOX17*-promoter repression	-ATTTGATTGCCCGCCTCAGA-	-GACGCTTTTGTTGGGCATGT-
NYNRIN	Aminopurvalanol-a	*SOX17*-promoter repression	-GCAGGAATGGTTCATGGTGC-	-GCACAGGCCCTTCAGGTATT-
LAMP3	Aminopurvalanol-a	*SOX17*-promoter repression	-CAAACATGCGGTGGTGATGT-	-GTGTAGTCAGACGAGCACTCA-
AURKA	YK-4279	*SOX17*-promoter activation	-GTGGCGGAGCGTCAAGT-	-AAATATCCCCGCACTCTGGC-
CNEPE	YK-4279	*SOX17*-promoter activation	-ACACGGATGCTGGTGACCTC-	-TGCCAAGGCACCAAGTAACTC-
DEPDC1	YK-4279	*SOX17*-promoter activation	-TGCCATGAAGTGCCTAGCAA-	-ACTGTGATGTAGCCACAAACAACC-
KIF20A	YK-4279	*SOX17*-promoter activation	-GCTTCGGCGACTAGGTGTGA-	-TTGCGTACCACAGACCCCAA-
	Aminopurvalanol-a	Signal-1 repression		

GABPB1	YK-4279	*SOX17*-promoter activation	-GACCTGGAGGGGTGGTGAAC-	-TCTGTGGCCACTACTGGAGTT-
SEMA4F	YK-4279	*SOX17*-promoter activation	-TCGCTTTATCCCTGCCCTTC-	-TGGACACATCCTCTTTCTTGCC-
CDC20	Aminopurvalanol-a	Signal-1 repression	-CAAGGAGCTCATCTCAGGCCA-	-GTCCAACTCAAAACAGCGCCA-
CCNA2	Aminopurvalanol-a	Signal-1 repression	-GCGCTGGCGGTACTGAAGTC-	-TGCTTTCCAAGGAGGAACGGT-
SORT1	Aminopurvalanol-a	Signal-1 repression	-CCGGGTCCGGGACTTC-	-GCTATCTCCAACCCAGGACAAG-
PLXNA3	Aminopurvalanol-a	Signal-1 repression	-CTCCATGGCTGGTGTCATTGTG-	-GCGTGTCTGAGGGGATCTTGA-
LAMB2	Aminopurvalanol-a	Signal-1 repression	-CAGACCCCTACAGCTCACGG-	-TGCCACGTACAACCAGCTCA-

Supplemental Table S8) Basic demographics of individuals who donated blood for derivation of endothelial colony forming cells.

Diagnosis	Sex	Age
Healthy volunteer	F	23
Healthy volunteer	M	22
Healthy volunteer	F	57
Healthy volunteer	F	36
Healthy volunteer	F	29
Healthy volunteer	F	50
1.1: Idiopathic PAH	M	80
1.1: Idiopathic PAH	F	52
1.1: Idiopathic PAH	M	51
1.1: Idiopathic PAH	F	60
1.1: Idiopathic PAH	F	59
1.1: Idiopathic PAH	M	79
1.1: Idiopathic PAH	F	79
1.1: Idiopathic PAH	M	62
1.1: Idiopathic PAH	F	72
1.1: Idiopathic PAH	F	70
1.1: Idiopathic PAH	F	78

UK National PAH Cohort Study Consortium

Marta Bleda, Charaka Hadinnapola, Matthias Haimel, Kate Auckland, Tobias Tilly, Jennifer M. Martin, Katherine Yates, Carmen M. Treacy, Margaret Day, Alan Greenhalgh, Debbie Shipley, Andrew J. Peacock, Val Irvine, Fiona Kennedy, Shahin Moledina, Lynsay MacDonald, Eleni Tamvaki, Anabelle Barnes, Victoria Cookson, Latifa Chentouf, Souad Ali, Shokri Othman, Lavanya Ranganathan, J. Simon R. Gibbs, Rosa DaCosta, Joy Pinguel, Natalie Dormand, Alice Parker, Della Stokes, Dipa Ghedia, Yvonne Tan, Tanaka Ngcozana, Ivy Wanjiku, Gary Polwarth, Rob V. Mackenzie Ross, Jay Suntharalingam, Mark Grover, Ali Kirby, Ali Grove, Katie White, Annette Seatter, Amanda Creaser-Myers, Sara Walker, Stephen Roney, Charles A. Elliot, Athanasios Charalampopoulos, Ian Sabroe, Abdul Hameed, Iain Armstrong, Neil Hamilton, Alex M. K. Rothman, Andrew J. Swift, James M. Wild, Florent Soubrier, Mélanie Eyries, Marc Humbert, David Montani, Barbara Girerd, Laura Scelsi, Stefano Ghio, Henning Gall, Ardi Ghofrani, Richard Trembath, Harm J. Bogaard, Anton Vonk Noordegraaf, Arjan C. Houweling, Anna Huis in't Veld & Gwen Schotte

Freeman Hospital, Newcastle, UK.

Golden Jubilee National Hospital, Glasgow, UK.

Great Ormond Street Hospital, London, UK.

Hammersmith Hospital, London, UK.

Royal Brompton Hospital, London, UK.

Royal Free Hospital, London, UK.

Royal United Bath Hospitals, Bath, UK.

Sheffield NIHR Clinical Research Facility, Royal Hallamshire Hospital, Sheffield, UK.

Département de génétique, hôpital Pitié-Salpêtrière, Assistance Publique-Hôpitaux de Paris, and UMR_S 1166-ICAN, INSERM, UPMC Sorbonne Universités, Paris, France.

Université Paris-Sud, Faculté de Médecine, Université Paris-Saclay, AP-HP, Centre de référence de l'hypertension pulmonaire sévère, INSERM UMR_S 999, Hôpital Bicêtre, Le Kremlin-Bicêtre, France.

San Matteo, Pavia, Italy.

University of Giessen, Giessen, Germany.

VU University Medical Center, Amsterdam, The Netherlands.
