# Supplementary material for: SOX17 enhancer variants disrupt transcription factor binding and enhancer inactivity drives pulmonary hypertension
Source: Circulation. Author manuscript; Available in PMC 2023 May 25. (PMC7614572; doi:10.1161/CIRCULATIONAHA.122.061940)

<u>Walters et al. 2023 CIRCULATIONAHA/2022/061940 : SOX17 enhancer function in pulmonary hypertension</u>

<u>Unedited gel images for all representative cropped gels in the manuscript</u>

Red boxes highlight the relevant lanes/areas.

Full unedited gels for **Figure 7B**:

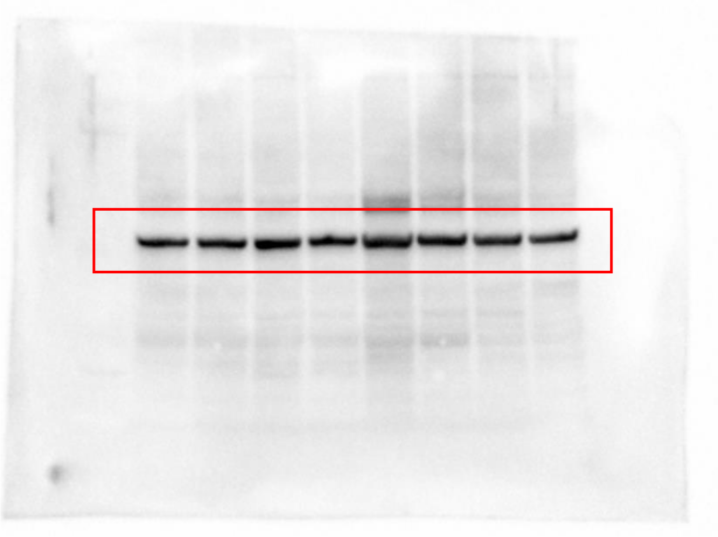

Actin

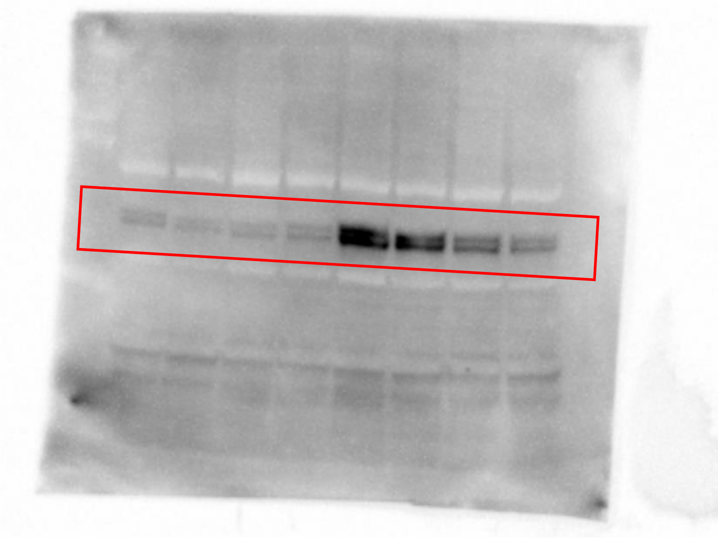

SOX17

Full unedited gels for supplemental **Figure S1E**:

These images had the colour inverted in editing.

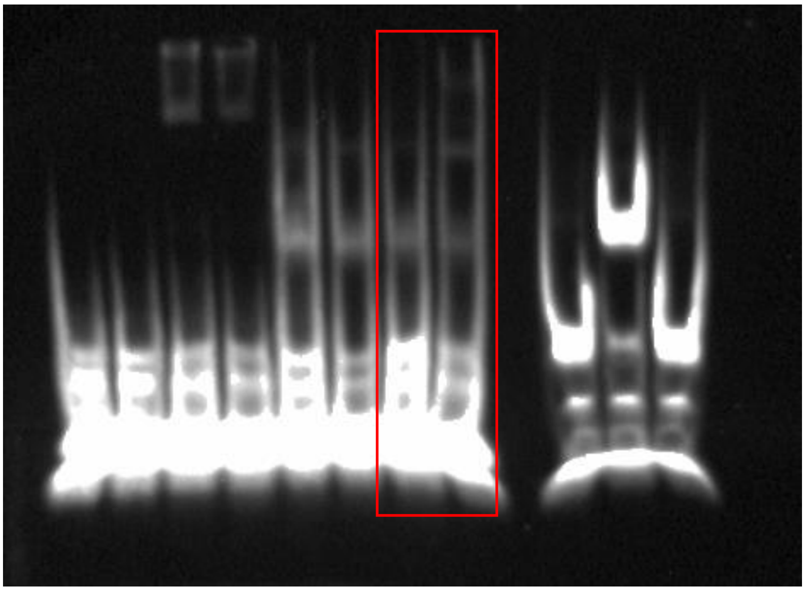

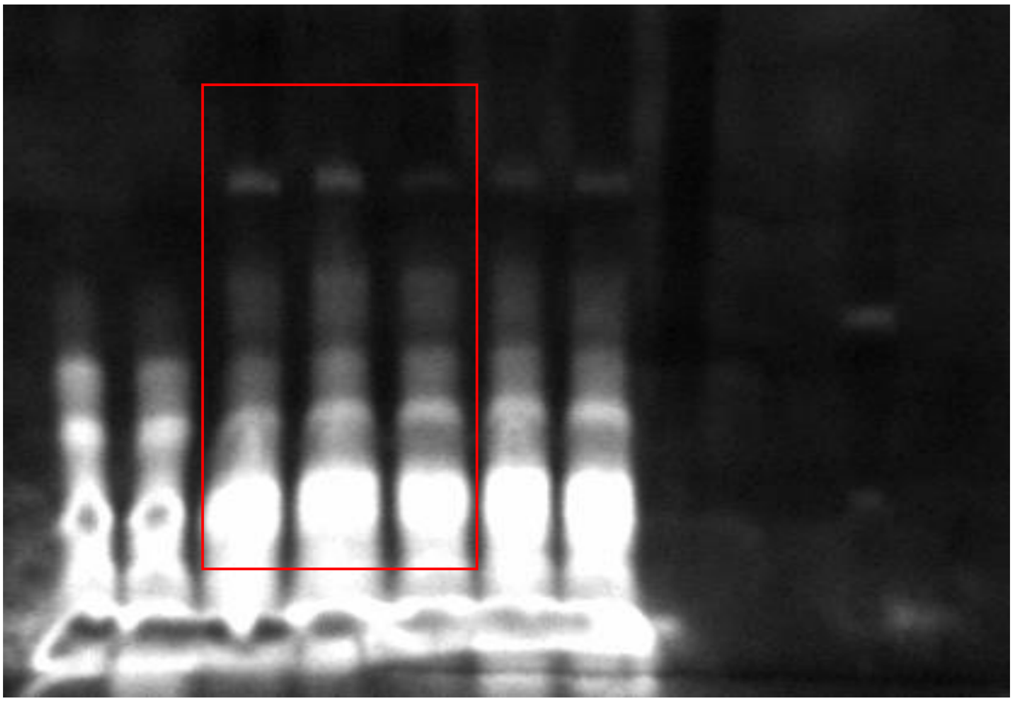

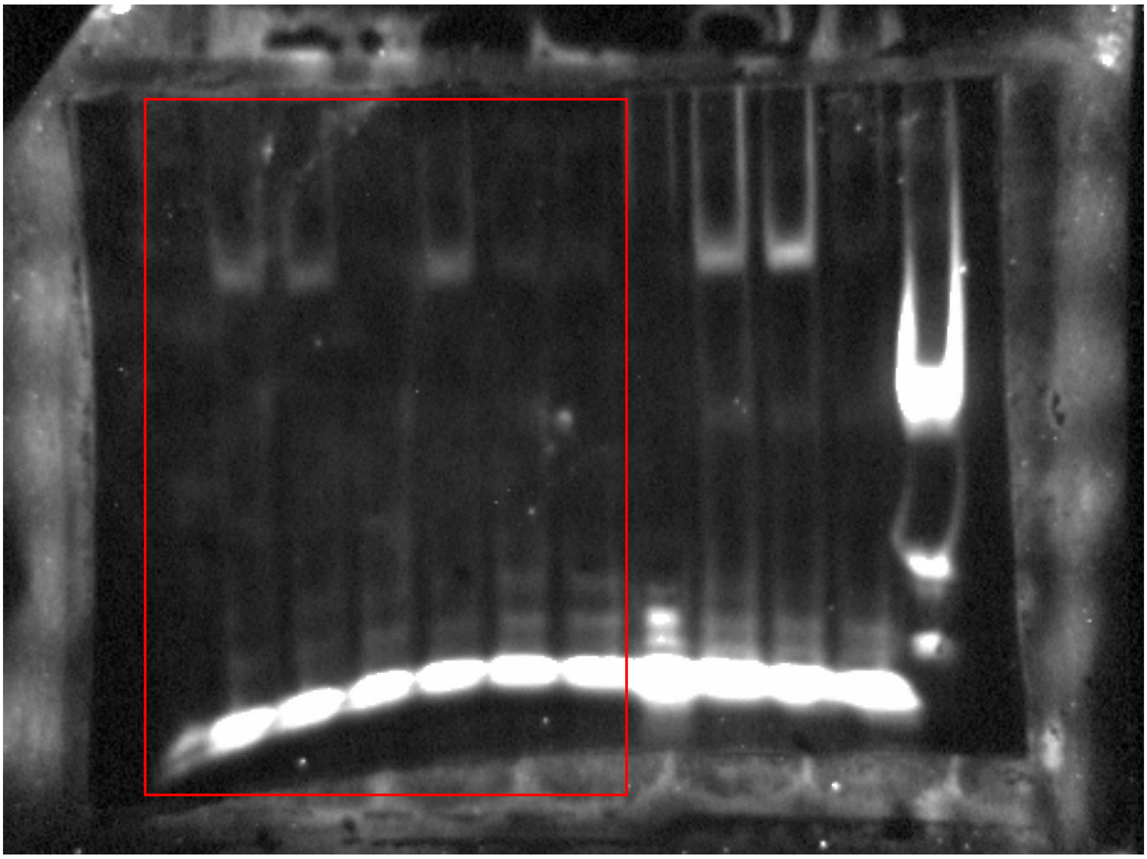

Full unedited gels for supplemental **Figure S1F**:

These images had the colour inverted in editing.

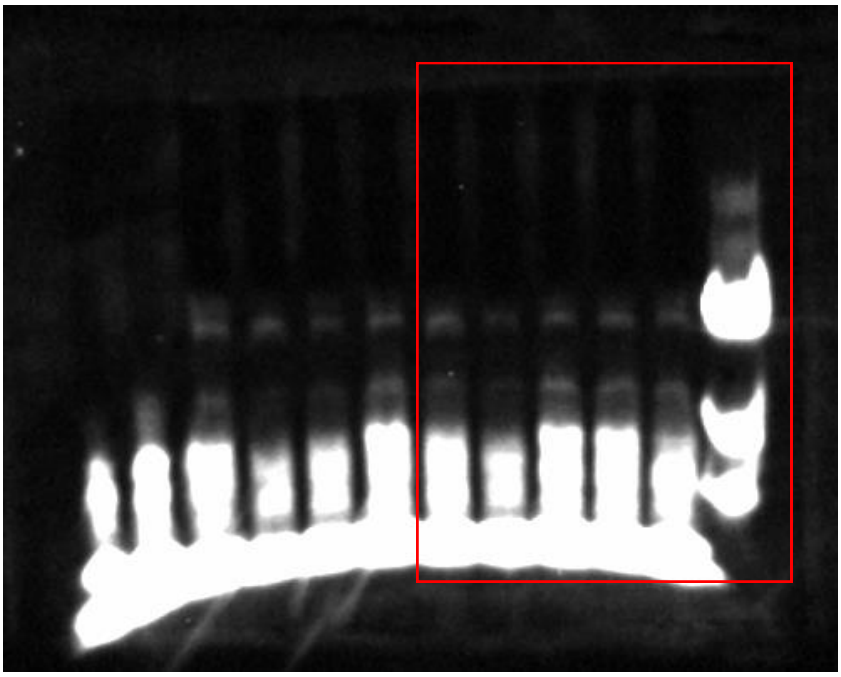

Full unedited gels for supplemental **Figure S2B**:

These gels were cut prior to blotting with primary antibodies. Top, Vincullin (115kDa). Bottom, Sox17 (44kDa).

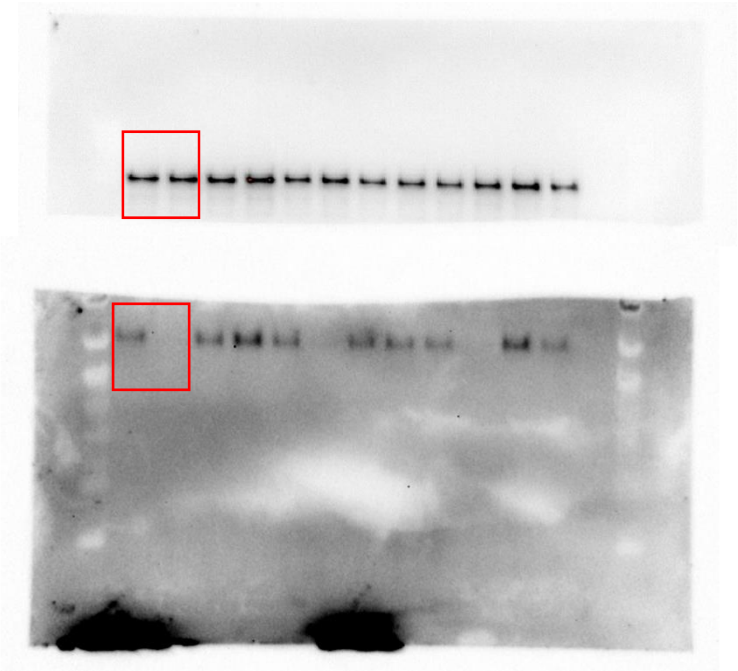

Supplement: Unedited gels [file EMS173250-supplement-Unedited_gels.pdf]
